# Pre-existing Liver Diseases and On-Admission Liver-Related Laboratory Tests in COVID-19: A Prognostic Accuracy Meta-Analysis With Systematic Review

**DOI:** 10.3389/fmed.2020.572115

**Published:** 2020-11-13

**Authors:** Szilárd Váncsa, Péter Jeno Hegyi, Noémi Zádori, Lajos Szakó, Nóra Vörhendi, Klementina Ocskay, Mária Földi, Fanni Dembrovszky, Zsuzsa Réka Dömötör, Kristóf Jánosi, Zoltán Rakonczay, Petra Hartmann, Tamara Horváth, Bálint Erőss, Szabolcs Kiss, Zsolt Szakács, Dávid Németh, Péter Hegyi, Gabriella Pár

**Affiliations:** ^1^Institute for Translational Medicine, Medical School, University of Pécs, Pécs, Hungary; ^2^János Szentágothai Research Center, University of Pécs, Pécs, Hungary; ^3^Doctoral School of Clinical Medicine, University of Szeged, Szeged, Hungary; ^4^Faculty of Medicine, George Emil Palade University of Medicine, Pharmacy, Science and Technology of Târgu Mureş, Târgu Mureş, Romania; ^5^Heart Institute, Medical School, University of Pécs, Pécs, Hungary; ^6^Department of Pathophysiology, University of Szeged, Szeged, Hungary; ^7^Institute of Surgical Research, University of Szeged, Szeged, Hungary; ^8^Division of Gastroenterology, First Department of Medicine, Medical School, University of Pécs, Pécs, Hungary

**Keywords:** SARS—CoV-2, COVID-19, prognosis, hepatology, pandemic (COVID-19)

## Abstract

**Background:** We aimed to perform a systematic search and meta-analysis to evaluate the prognostic value of on-admission liver function tests and pre-existing liver diseases on the clinical course of coronavirus disease 2019 (COVID-19).

**Methods:** The study was registered on PROSPERO (CRD42020182902). We searched five databases between 01/01/2020 and 04/23/2020. Studies that reported on liver-related comorbidities and/or laboratory parameters in patients with COVID-19 were included. The main outcomes were COVID-19 severity, intensive care unit (ICU) admission, and in-hospital mortality. Analysis of predictive models hierarchical summary receiver-operating characteristic (HSROC) was conducted with a 95% confidence interval (CI).

**Results:** Fifty studies were included in the meta-analysis. High specificity was reached by acute liver failure associated by COVID-19 (0.94, 95% CI: 0.71–0.99) and platelet count (0.94, 95% CI: 0.71–0.99) in the case of mortality; chronic liver disease (CLD) (0.98, 95% CI: 0.96–0.99) and platelet count (0.82, 95% CI: 0.72–0.89) in the case of ICU requirement; and CLD (0.97, 95% CI: 0.95–0.98), chronic hepatitis B infection (0.97, 95% CI: 0.95–0.98), platelet count (0.86, 95% CI: 0.77–0.91), and alanine aminotransferase (ALT) (0.80, 95% CI: 0.66–0.89) and aspartate aminotransferase (AST) (0.84, 95% CI: 0.77–0.88) activities considering severe COVID-19. High sensitivity was found in the case of C-reactive protein (CRP) for ICU requirement (0.92, 95% CI: 0.80–0.97) and severe COVID-19 (0.91, 95% CI: 0.82–0.96).

**Conclusion:** On-admission platelet count, ALT and AST activities, CRP concentration, and the presence of acute and CLDs predicted the severe course of COVID-19. To highlight, pre-existing liver diseases or acute liver injury associated by severe acute respiratory syndrome coronavirus 2 (SARS-CoV-2) infection plays an important role in the prediction of mortality.

## Introduction

In December 2019, a local outbreak of pneumonia caused by a novel coronavirus, namely, severe acute respiratory syndrome coronavirus 2 (SARS-CoV-2), was detected in Wuhan, China. In most cases, coronavirus disease 2019 (COVID-19) is an acute, self-limiting disease with a relatively brief period of symptoms and resolution within days. However, it can reach in-hospital mortality of 3–7% (1), which can result from massive alveolar damage, consequential acute respiratory distress syndrome (ARDS), respiratory failure, septic shock, or multiple organ dysfunction (2, 3).

It is important to explore the prognostic factors, which have a significant impact on the disease course, given the rapid spread of COVID-19 and its high mortality rate. The detrimental effects of hypertension, cardiovascular diseases, kidney disease, and diabetes mellitus on the disease course are already proven (4–6). Due to the limited number of reports on COVID-19 with underlying chronic liver disease (CLD) to date, the impact of pre-existing liver pathologies on COVID-19 progression and outcomes is unknown.

Although coronaviruses cause the worst damage on the lungs, studies suggest that other organs, such as the liver, intestines, heart, and central nervous system, could also be affected (7–11). In COVID-19, almost half of the hospitalized patients have various degrees of liver test abnormalities, and liver impairment was also observed in 14–53% of the patients (12).

We aimed to appraise the currently available literature of confirmed SARS-CoV-2 infections critically and to investigate the prognostic value of on-admission liver function and liver conditions on the clinical course of COVID-19.

## Materials and Methods

Our systematic review and meta-analysis was planned and reported according to the PRISMA (Preferred Reporting Items for Systematic Reviews and Meta-Analyses) 2009 Statement (13) (Supplementary Table 1). This study was registered in advance on PROSPERO under registration number CRD42020182902 (see https://www.crd.york.ac.uk/prospero).

### Search and Selection

A systematic search was conducted by two independent reviewers (LS and NZ) to identify all the relevant records on the prognostic value of liver impairment in COVID-19 patients published from January 1, 2020 to April 23, 2020. The search was performed in MEDLINE via PubMed, Embase, Scopus, Cochrane Library, and Web of Science with the terms (“covid 19”) OR (“Wuhan virus”) OR (“coronavirus”) OR (“2019 nCoV”) OR (“SARS-CoV-2”) without language or other restrictions. References were managed by the EndNote X9 software (Clarivate Analytics, Philadelphia, PA, USA). Following the removal of duplicates, title and abstract screening were performed by two independent reviewers (PJH and NV) to identify potentially eligible articles. Disagreements were reviewed by a third review author (KJ) and resolved by consensus. The reference lists of the relevant articles were hand-searched, and additional eligible records were included.

We included studies without any restriction that reported on (*C*) liver diseases (as defined by eligible studies) and/or on-admission liver function tests in (*P*) patients with confirmed COVID-19. Concerning the laboratory parameters, cut-off values predefined by the individual studies were used for abnormal parameters (*O*). The assessed outcomes were as follows: in-hospital mortality, severe SARS-CoV-2 infection defined by eligible studies, and intensive care unit (ICU) requirement defined by eligible studies. Severity of COVID-19 was classified according to the guidelines on the Diagnosis and Treatment of COVID-19 issued by the National Health Commission of China (14). Details are presented in Supplementary Table 2. Studies with a sample size of fewer than 15 subjects were excluded because of the small effect size. When there were multiple publications using data with overlapping study populations, we included the one with a greater sample size.

### Data Extraction and Outcomes

Relevant data were independently extracted from studies by review authors ZRD and FD. These included: first author, year of publication, country of origin, time interval and place of the study, study design, basic characteristics of the study population (age, percentage of females, and size of the study groups), the proportion of event (in-hospital mortality, severe SARS-CoV-2 infection, and need for ICU care) in patients with and without liver impairment, time of measurement for outcomes, and serum laboratory parameters [total bilirubin, albumin, aspartate aminotransferase (AST), alanine aminotransferase (ALT), alkaline phosphatase (ALP), gamma-glutamyl transferase (GGT), platelet count, international normalized ratio (INR), lactate dehydrogenase (LDH), and C-reactive protein (CRP)], predefined cut-off values, and information for risk of bias assessment. Extracted data were validated by MF and SK.

### Statistical Analysis

Calculations were performed by Stata 15 data analysis and statistical software (StataCorp LLC, College Station, TX, USA). The first preference was the analysis of hierarchical summary receiver-operating characteristic (HSROC) predictive models with 95% confidence interval (CI) when at least five articles were available for the given outcome. The area under the curve (AUC) values and their 95% CIs for each prognostic factor and outcome were collected, and a meta-analysis using the random effect model to gain pooled AUC estimates with 95% CI was performed. Second preference in case of dichotomous variables (mortality, severe vs non-severe, and ICU vs. non-ICU) was the calculation of odds ratios (OR) with a 95% CI. A *p* < 0.05 was considered statistically significant.

Heterogeneity was tested with *I*^2^ and χ^2^ tests. As suggested by the Cochrane Handbook, *I*^2^ values were interpreted as moderate (30–60%), substantial (50–90%), and considerable (75–100%) heterogeneity (15). A *p* < 0.10 was considered significant. Forest plots and HSROC curves were used to present the results of the meta-analyses. Publication bias was checked by Egger's test (alpha = 0.1) when at least 10 studies were available (16). A *p* < 0.1 was chosen because of the low number of studies included in our analyses, since it can determine a significant heterogeneity with greater certainty (17).

### Assessment of Risk of Bias

Bias assessment was performed by two authors independently (PHa and TH) using the modified Quality In Prognosis Studies (QUIPS) assessment tool (18). Disagreements were resolved by a third investigator (GP). Details of the used QUIPS tool are shown in the footnote of Supplementary Table 5.

### Protocol Deviation

We waived the need for data extraction and analysis regarding the continuous variables and Funnel plots after statistical consultation as it did not provide additional value.

## Results

**Figure 1:**
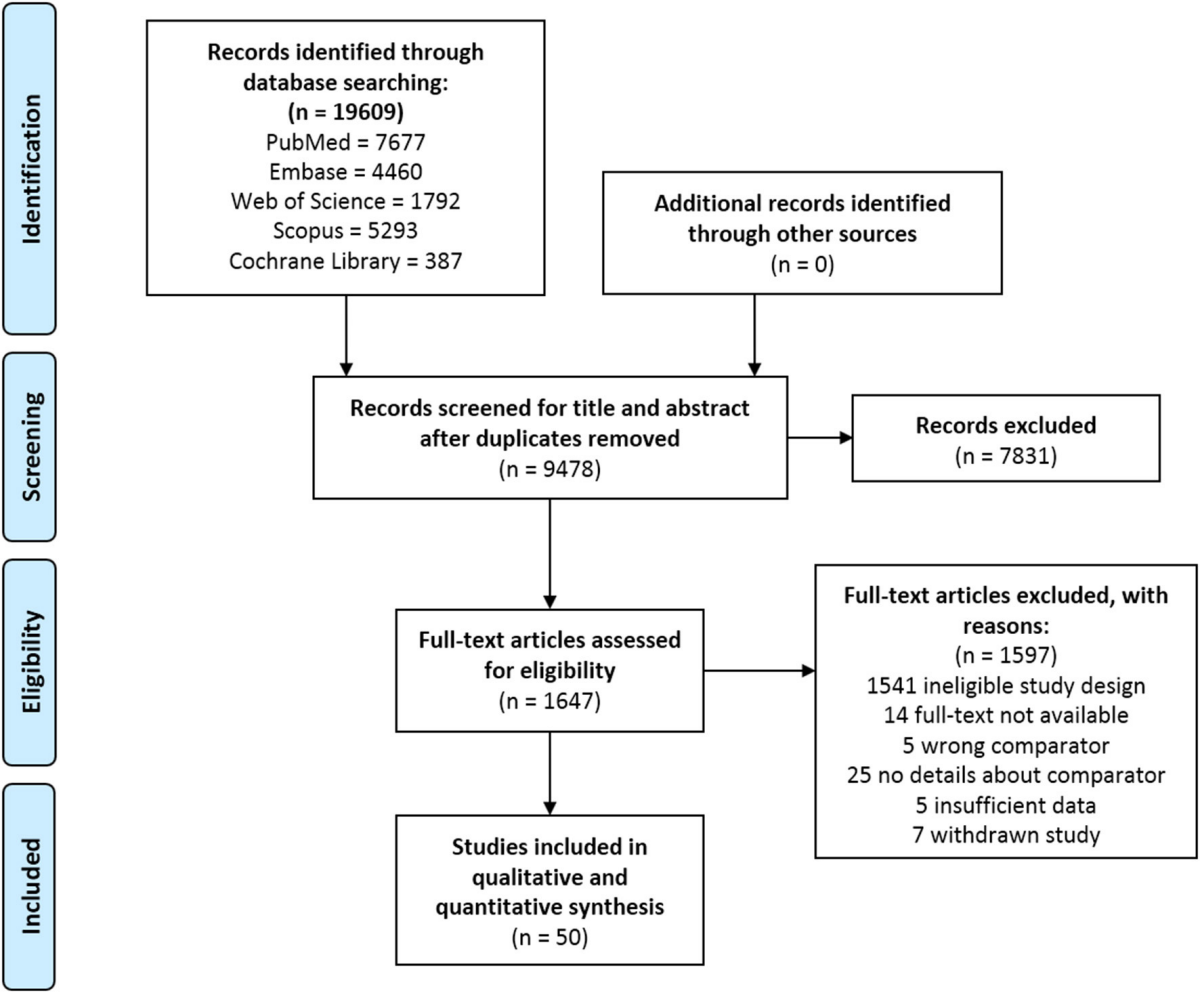
PRISMA flowchart.

Overall, 19,609 records were identified through the comprehensive search, from which 1,647 full texts were reviewed, and 50 studies were included in the qualitative and quantitative syntheses. The selection process is presented in Figure 1.

Basic characteristics of the included studies are shown in Table 1 and Supplementary Table 3. Detailed eligibility criteria for each included study are presented in Supplementary Table 4.

**Table 1:** Basic characteristics of the included studies.

**Study**	**Country**	**Cohort type**	**Total number of patients (female %)**	**Age (year)^**‡**^**	**Outcome(s)**	
					**Definition**	**Event number (event rate %)**
Cai et al. (19)	China	Retrospective	298 (51)	48	Severe COVID-19	58 (19)
Cai et al. (20)	China	Retrospective	318 (NR)	NR	Severe COVID-19	85 (27)
Cao et al. (21)	China	Prospective	102 (48)	54	Mortality	17 (17)
Chen et al. (22)	China	Retrospective	21 (19)	56	Severe COVID-19	11 (52)
Chen et al. (4)	China	Retrospective	1,590 (43)	NR	Mortality	50 (3)
Chen et al. (23)	China	Retrospective	274 (38)	62	Mortality	113 (41)
Chen et al. (24)	China	Retrospective	203 (38)	74	Mortality	19 (9)
Chen et al. (25)	China	Retrospective	48 (23)	65	ICU admission	17 (35)
Colombi et al. (26)	Italy	Retrospective	236 (25)	68	ICU admission	108 (46)
Du et al. (27)	China	Retrospective	109 (32)	71	ICU admission	51 (47)
Fan et al. (28)	Singapore	Retrospective	67 (45)	42	ICU admission	9 (13)
Fan et al. (29)	China	Retrospective	148 (NR)	NR	Mortality	1 (1)
					ICU admission	10 (7)
Feng et al. (30)	China	Retrospective	476 (43)	53	ICU admission	70 (15)
Goyal et al. (31)	USA	Retrospective	393 (39)	62	ICU admission	130 (33)
Grein et al. (32)	Multiple^†^	Retrospective	53 (25)	64	ICU admission	34 (64)
Guan et al. (5)	China	Retrospective	1,099 (42)	47	Severe COVID-19	173 (16)
Guan et al. (33)	China	Retrospective	1,590 (43)	49	ICU admission	99 (6)
					Severe COVID-19	254 (16)
Huang et al. (34)	China	Prospective	41 (27)	49	ICU admission	13 (32)
Ji et al. (35)	China	Retrospective	202 (44)	45	Severe COVID-19	39 (19)
Ji et al. (36)	China	Retrospective	208 (44)	44	Severe COVID-19	40 (19)
Li et al. (37)	China	Retrospective	548 (49)	60	Severe COVID-19	269 (49)
Liu et al. (38)	China	Retrospective	383 (58)	46	Mortality	49 (13)
Qi et al. (39)	China	Prospective	70 (NR)	NR	Severe COVID-19	3 (4)
Qian et al. (40)	China	Retrospective	324 (49)	51	Severe COVID-19	26 (8)
Qin et al. (41)	China	Retrospective	452 (48)	58	Severe COVID-19	286 (63)
Richardson et al. (42)	USA	Retrospective	2,634 (NR)	NR	Mortality	553 (21)
Ruan et al. (43)	China	Retrospective	150 (32)	NR	Mortality	68 (45)
Shen et al. (44)	China	Retrospective	119 (53)	49	Severe COVID-19	20 (17)
Shi et al. (45)	China	Retrospective	487 (47)	46	Severe COVID-19	49 (10)
To et al. (46)	China	Retrospective	23 (43)	62	Severe COVID-19	10 (43)
Tu et al. (47)	China	Retrospective	174 (55)	NR	Mortality	25 (14)
Wan et al. (48)	China	Retrospective	135 (47)	47	Severe COVID-19	40 (30)
Wan et al. (49)	China	Retrospective	123 (46)	NR	Severe COVID-19	21 (17)
Wang et al. (50)	China	Retrospective	339 (51)	69	Mortality	65 (19)
Wang et al. (51)	China	Retrospective	55 (60)	49	Severe COVID-19	2 (4)
Wang et al. (52)	China	Retrospective	69 (54)	42	ICU admission	14 (20)
Wu et al. (53)	China	Retrospective	280 (46)	43	ICU admission	83 (30)
Yang et al. (54)	China	Retrospective	93 (40)	46	Severe COVID-19	24 (26)
Yang et al. (55)	China	Retrospective	1,476 (47)	57	Mortality	238 (16)
Yang et al. (56)	China	Retrospective	52 (33)	60	Mortality	32 (62)
Zhang et al. (57)	China	Retrospective	221 (51)	55	Severe COVID-19	55 (25)
Zhang et al. (58)	China	Retrospective	663 (52)	56	Mortality	25 (4)
Zhang et al. (59)	China	Retrospective	140 (49)	57	Severe COVID-19	58 (41)
Zhang et al. (60)	China	Retrospective	120 (64)	45	Severe COVID-19	30 (25)
Zhang et al. (61)	China	Retrospective	115 (57)	50	Severe COVID-19	31 (27)
Zheng et al. (62)	China	Retrospective	161 (50)	45	Severe COVID-19	30 (19)
Zheng et al. (63)	China	Retrospective	96 (40)	55	Severe COVID-19	74 (77)
Zhou et al. (64)	China	Retrospective	191 (38)	56	Mortality	54 (28)
Zhou et al. (65)	China	Retrospective	15 (33)	62	Mortality	7 (47)
Zhou et al. (66)	China	Retrospective	21 (38)	66	ICU admission	13 (62)

*COVID-19, coronavirus disease 2019; ICU, intensive care unit admission; NR, not reported*.

Multiple countries (USA, Japan, Italy, Austria, France, Germany, Netherlands, Spain, and Canada);

‡ *mean or median*.

### Diagnostic Metrics

For the prediction of mortality, a high specificity was reached by liver failure (specificity: 0.94, 95% CI: 0.71–0.99) and platelet count (specificity: 0.94, 95% CI: 0.71–0.99) and a moderate sensitivity by LDH (sensitivity: 0.81, 95% CI: 0.78–0.85).

For the prediction of possible ICU requirement, CLD (specificity: 0.98, 95% CI: 0.96–0.99) and platelet count (specificity: 0.82, 95% CI: 0.72–0.89) proved to be specific, whereas CRP was associated with high sensitivity (sensitivity: 0.92, 95% CI: 0.80–0.97).

For the prediction of severe disease course, CLD (specificity: 0.97, 95% CI: 0.95–0.98) and chronic hepatitis B infection (specificity: 0.97, 95% CI: 0.95–0.98) were highly specific, and platelet count (specificity: 0.86, 95% CI: 0.77–0.91), ALT (specificity: 0.80, 95% CI: 0.66–0.89), and AST (specificity: 0.84, 95% CI: 0.77–0.88) were moderately specific, whereas high sensitivity was reached by CRP (sensitivity: 0.91, 95% CI: 0.82–0.96).

CLD for mortality and total bilirubin in case of severe COVID-19 could not be analyzed because it was not feasible despite the number of included studies.

Detailed results about the AUC, sensitivity, specificity, likelihood ratios, and heterogeneity are shown in Table 2. The HSROC curves are summarized in Supplementary Figures 1–3.

**Table 2:** Summary table of mortality, severe COVID-19, and intensive care unit requirement based on the HSROC analysis.

**Prognostic factor**	**No. of studies** **(no. of cases)**	**AUC** **(95% CI)**	**Sensitivity** **(95% CI)**	***I*^**2**^ (%)**	**Chi^**2**^**	**Specificity** **(95% CI)**	***I*^**2**^ (%)**	**Chi^**2**^**	**PLR** **(95% CI)**	**NLR** **(95% CI)**
**Mortality**										
Liver failure	5 (3,523)	0.67 (0.63–0.71)	0.31 (0.12–0.59)	99	0.001	0.94 (0.71–0.99)	99	0.001	5.5 (1.6–19.4)	0.73 (0.55–0.97)
Platelet count	5 (3,259)	0.71 (0.67–0.75)	0.40 (0.23–0.59)	95	0.001	0.89 (0.75–0.96)	99	0.001	3.7 (1.5–9)	0.68 (0.5–0.91)
ALT	5 (2,127)	0.76 (0.72–0.79)	0.41 (0.30–0.53)	71	0.01	0.77 (0.75–0.80)	0	0.63	1.8 (1.4–2.4)	0.76 (0.64–0.92)
LDH	5 (2,149)	0.81 (0.78–0.85)	0.87 (0.74–0.94)	71	0.01	0.58 (0.41–0.73)	95	0.001	2.1 (1.4–3.1)	0.22 (0.1–0.48)
**Intensive care unit requirement**										
Chronic liver disease	5 (831)	0.80 (0.77–0.84)	0.03 (0.01–0.06)	0	0.48	0.98 (0.96–0.99)	59	0.04	1.3 (0.5–3.3)	0.99 (0.97–1.02)
Platelet count	5 (628)	0.47 (0.43–0.52)	0.18 (0.11–0.28)	35	0.19	0.82 (0.72–0.89)	63	0.03	1 (0.6–1.6)	1 (0.9–1.12)
ALT	5 (1,190)	0.58 (0.54–0.62)	0.32 (0.25–0.41)	33	0.20	0.76 (0.70–0.81)	52	0.08	1.3 (1.1–1.7)	0.89 (0.81–0.98)
AST	6 (1,229)	0.65 (0.61–0.69)	0.55 (0.47–0.62)	37	0.16	0.69 (0.62–0.75)	78	0.001	1.7 (1.5–2.1)	0.66 (0.57–0.76)
CRP	6 (1,412)	0.75 (0.72–0.79)	0.92 (0.80–0.97)	88	0.001	0.31 (0.14–0.54)	95	0.001	1.3 (1.1–1.7)	0.27 (0.16–0.46)
**Severe COVID-19**										
Chronic liver disease	10 (2,182)	0.65 (0.60–0.69)	0.03 (0.02–0.07)	75	0.001	0.97 (0.95–0.98)	76	0.001	1.2 (0.6–2.1)	1 (0.97–1.02)
Chronic hepatitis B	7 (3,911)	0.71 (0.67–0.75)	0.03 (0.01–0.08)	84	0.001	0.97 (0.95–0.98)	85	0.001	1.2 (0.6–2.4)	1 (0.97–1.02)
Platelet count	7 (1,868)	0.66 (0.62–0.70)	0.26 (0.15–0.42)	88	0.001	0.86 (0.77–0.91)	92	0.001	1.8 (1.2–2.7)	0.86 (0.75–0.99)
ALT	8 (1,625)	0.60 (0.55–0.64)	0.31 (0.19–0.48)	94	0.001	0.80 (0.66–0.89)	96	0.001	1.6 (1.1–2.2)	0.86 (0.74–0.99)
AST	9 (2,780)	0.70 (0.65–0.74)	0.40 (0.30–0.50)	88	0.001	0.84 (0.77–0.88)	90	0.001	2.4 (1.8–3.2)	0.72 (0.63–0.83)
LDH	9 (2,500)	0.75 (0.71–0.79)	0.67 (0.57–0.77)	93	0.001	0.72 (0.62–0.80)	95	0.001	2.4 (1.8–3.1)	0.45 (0.35–0.58)
CRP	6 (2,253)	0.68 (0.64–0.72)	0.91 (0.82–0.96)	89	0.001	0.34 (0.23–0.47)	94	0.001	1.4 (1.2–1.5)	0.27 (0.18–0.42)

*COVID-19, coronavirus disease 2019; HSROC, hierarchical summary receiver-operating characteristic; ALT, alanine aminotransferase; AST, aspartate aminotransferase; AUC, area under the curve; CI, confidence interval; CRP, C-reactive protein; I^2^ and Chi^2^, heterogeneity; LDH, lactate dehydrogenase; NLR, negative likelihood ratio; PLR, positive likelihood ratio*.

### Analysis of the Strength of the Association

Liver failure (OR: 7.59; 95% CI: 1.84–31.30), platelet count (OR: 5.36; 95% CI: 1.28–22.37), albumin level (OR: 6.32; 95% CI: 1.40–28.60), and ALT (OR: 2.49; 95% CI: 1.75–3.56), AST (OR: 5.39; 95% CI: 3.67–7.91), and LDH (OR: 9.23; 95% CI: 2.56–33.31) activities were related to a high rate of mortality. CLD, hepatitis B infection, and CRP concentration did not show significant difference, considering mortality.

Albumin (OR: 3.79; 95% CI: 2.08–6.93), ALT (OR: 1.56; 95% CI: 1.61–2.11), AST (OR: 2.53; 95% CI: 1.92–3.35), and LDH (OR: 7.95; 95% CI: 4.54–13.92) levels and CRP (OR: 4.72; 95% CI: 2.59–8.58) concentration were accompanied with high rate of ICU admission. A significant difference could not be stated regarding the need for ICU considering CLD, liver dysfunction, and platelet count.

Fatty liver disease (OR: 3.86; 95% CI: 1.20–12.47), liver failure (OR: 3.27; 95% CI: 1.20–8.87), total bilirubin (OR: 1.89; 95% CI: 1.35–2.63), platelet count (OR: 2.34; 95% CI: 1.53–3.58), albumin level (OR: 3.11; 95% CI: 1.61–6.01), ALT (OR: 1.82; 95% CI: 1.18–2.81), AST (OR: 3.34; 95% CI: 2.37–4.71), LDH (OR: 5.02; 95% CI: 3.41–7.40), CRP (OR: 4.52; 95% CI: 3.16–6.49), and GGT (OR: 3.03; 95% CI: 1.60–5.7) were accompanied with a higher risk for more severe course. CLD, hepatitis B infection, and elevated level of ALP did not show significant difference concerning severity.

Results of the analysis of association and heterogeneity are presented in Table 3. Forest plots for each analysis are shown in Supplementary Figures 4–17.

**Table 3:** Summary of findings.

**Prognostic factor**	**Mortality**				**Intensive care unit requirement**				**Severe COVID-19**			
	**No. of studies** **(no. of pts)**	**Odds ratio** **(95% CI)**	***I*^**2**^ (%)**	**Chi^**2**^**	**No. of studies** **(no. of pts)**	**Odds ratio** **(95% CI)**	***I*^**2**^ (%)**	**Chi^**2**^**	**No. of studies** **(no. of pts)**	**Odds ratio** **(95% CI)**	***I*^**2**^ (%)**	**Chi^**2**^**
Chronic liver disease	4 (646)^†^	1.5 (0.42–5.41)	0	0.54	5 (831)	1.42 (0.56–3.63)	0	0.72	10 (2,182)	1.45 (0.87–2.42)	0	0.7
Liver dysfunction	2 (145)	1.13 (0.36–3.58)	0	0.33	2 (384)	1.77 (0.62–5.06)	0	0.98	2 (163)	1.11 (0.36–3.47)	0	0.56
Chronic hepatitis B	2 (1,864)	1.18 (0.42–3.34)	0	0.97	1 (1,590)	0.55 (0.07–4.11)	NR	NR	7 (3,911)	1,55 (0.85–2.83)	13	0.33
Fatty liver disease	NR	NR	NR	NR	NR	NR	NR	NR	4 (964)	3.86 (1.2–12.47)^*^	79	0
Liver failure	5 (3,523)	7.59 (1.84–31.30)^*^	91	0	1 (43)	1.88 (0.47–7.54)	NR	NR	4 (1,185)	3.27 (1.2–8.87)^*^	70	0.02
Total bilirubin	1 (975)	5 (2.48–10.07)^*^	NR	NR	2 (395)	1.66 (0.45–6.06)	33	0.22	6 (2,059)	1.89 (1.35–2.63)^*^	0	0.57
Platelet count	5 (3,259)	5.36 (1.28–22.37)^*^	95	0	5 (628)	0.95 (0.63–1.44)	0	0.79	7 (1,868)	2.34 (1.53–3.58)^*^	46	0.09
International normalized ratio	NR	NR	NR	NR	1 (20)	5 (0.18–139.17)	NR	NR	1 (115)	0.72 (0.31–1.66)	NR	NR
Albumin	3 (944)	6.32 (1.4–28.6)^*^	63	0.07	3 (744)	3.79 (2.08–6.93)^*^	0	0.81	4 (1,205)	3.11 (1.61–6.01)^*^	69	0.02
Alanine aminotransferase	5 (2,127)	2.49 (1.75–3.56)^*^	10	0.35	5 (1,190)	1.56 (1.16–2.11)^*^	0	0.99	8 (1,625)	1.82 (1.18–2.81)^*^	70	0
Aspartate aminotransferase	4 (1,966)	5.39 (3.67–7.91)^*^	0	0.63	6 (1,229)	2.53 (1.92–3.35)^*^	0	0.48	9 (2,780)	3.34 (2.37–4.71)^*^	60	0.01
Lactate dehydrogenase	5 (2,149)	9.23 (2.56–33.31)^*^	85	0	4 (748)	7.95 (4.54–13.92)^*^	0	0.75	9 (2,500)	5.02 (3.41–7.4)^*^	66	0
C-reactive protein	4 (1,846)	9.19 (0.84–100.63)	77	0	6 (1,412)	4.72 (2.59–8.58)^*^	35	0.17	6 (2,253)	f4.52 (3.16–6.49)^*^	31	0.21
Alkaline phosphatase	NR	NR	NR	NR	1 (19)	0.11 (0–2.73)	NR	NR	4 (623)	1.71 (0.66–4.46)	24	0.27
Gamma-glutamyl transferase	NR	NR	NR	NR	1 (19)	1.39 (0.22–8.92)	NR	NR	3 (635)	3.03 (1.6–5.72)^*^	50	0.14

*CI, confidence interval; COVID-19, coronavirus disease 2019; I^2^ and Chi^2^, heterogeneity; NR, not reported*.

* *p < 0.05; one study could not be included in the analysis, because there were no events*.

### Risk of Bias Assessment

Results of the risk of bias assessment between studies are shown in Supplementary Table 5.

The assessment of publication bias could only be performed in the case of CLD on severe COVID-19. It did not suggest the presence of publication bias (*p* = 0.764).

## Discussion

**Figure 2:**
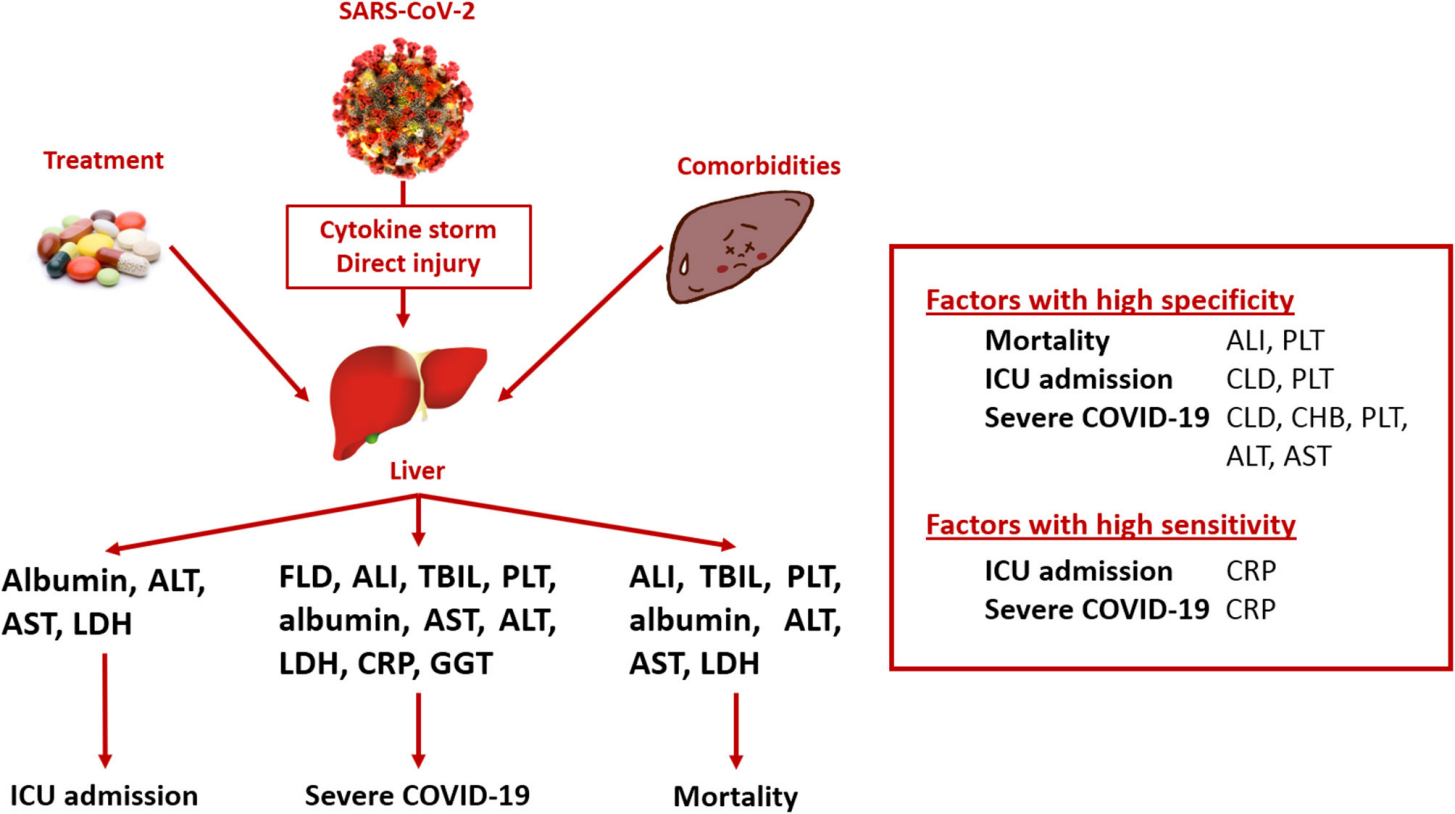
Summary of findings. ALI, acute liver injury; ALT, alanine aminotransferase; AST, aspartate aminotransferase; COVID-19, coronavirus disease 2019; CRP, C-reactive protein; FLD, fatty liver disease; GGT, gamma-glutamyl transferase; ICU, intensive care unit; LDH, lactate dehydrogenase; SARS-CoV-2, severe acute respiratory syndrome coronavirus 2; TBIL, total bilirubin.

This meta-analysis aimed to investigate the association between pre-existing liver diseases and on-admission liver functions and outcomes in COVID-19 infection, focusing on mortality, ICU admission, and severe disease course (Figure 2). Considering the prediction of mortality, liver failure and platelet count are highly specific, whereas LDH is moderately sensitive. For the prediction of ICU requirement, CLD was associated with high specificity, platelet count with moderate specificity, and CRP with high sensitivity. Regarding severe disease course, CLD and chronic hepatitis B infection were proven to be highly specific, and platelet count and ALT and AST activities were moderately specific, whereas CRP was highly sensitive.

In relation to the investigated factors and poorer outcomes, acute liver failure; platelet count; albumin level; ALT, AST, and LDH activities; and CRP concentration were associated with higher mortality. Albumin, ALT, AST, LDH, and CRP influenced the admission to the ICU. Fatty liver disease, liver injury, total bilirubin, ALT, AST, LDH, CRP, GGT, platelet count, and albumin level were associated with more severe disease course.

The knowledge about the impact of liver-related comorbidities in the clinical outcome of COVID-19 is limited. In line with our results, an earlier meta-analysis concluded that CLD is not associated with severity or mortality (67). However, clinicians should be skeptical about it, because these patients are more prone to infection due to cirrhosis-associated immune dysfunction and are more likely to have poor outcomes from ARDS (68, 69). This may account for the relatively low baseline prevalence of CLD in the included patients, as one previous meta-analysis suggests (70), or it was not well-reported. Further on, in a recently published letter on the involvement of the liver in COVID-19, the authors found an increased odds of severe infection and mortality in patients with liver injury (71). Another study analyzed the frequency of abnormal liver function derangements in severe COVID-19 and concluded that hypoalbuminemia followed by derangements in GGT and aminotransferases were more frequent in severe disease (72). On the other hand, another study highlights that digestive symptoms and liver injury are not uncommon in patients with SARS-CoV-2 infection (73).

Dysregulated hepatic immune responses caused by metabolic associated fatty liver disease (MAFLD) may contribute to cytokine storm in younger patients (74), whereas chronic low grade inflammation known to be associated with MAFLD may worsen outcome. Post-mortem liver biopsy showed overactivation of T cells in the liver, and liver injury is likely mediated by immune response rather than direct cytopathic damage (35).

Compared with previous results (12, 75, 76), our study reasserts that in severe forms of COVID-19, alterations of on-admission level of the liver enzymes can be observed, probably due to the virally induced cytotoxic T cells and the innate immune response against the virus. Another reason behind the liver test abnormalities in COVID-19 patients could be the cholangiocyte dysfunction due to direct infection of bile duct cells via angiotensin-converting enzyme 2 receptor (8). However, according to our results, ALP does not seem to be a significant predictive marker in COVID-19. Additionally, moderate microvesicular steatosis, mild lobular, and portal activity can be observed in the pathological samples of patients who died from COVID-19 (77).

Despite the lack of coagulation factors in liver diseases, a hypercoagulable state could also be present in COVID-19. A recent study concluded that COVID-19 disease has prominent manifestations from the hematopoietic system and is often associated with a major blood hypercoagulability (78). In histopathological findings, it was highlighted that extensive vascular portal and sinusoidal thrombosis could lead to abnormal high level of transaminases (79).

Considering the strengths of our meta-analysis, a rigorous methodology was followed. To our knowledge, this is the first study that addresses the prognostic value of on-admission liver parameters, underlying liver comorbidities, and COVID-19 induced hepatic failure on the level of sensitivity and specificity. On the other hand, our study has several limitations. We only included cohort studies that mostly originate from Asia, which might carry a high risk of bias. The definitions of the investigated outcomes were not uniform among the included reports; to estimate this problem, we applied a modified QUIPS. The cut-off values of laboratory parameters and the definition of liver diseases (Supplementary Tables 6, 7) were also slightly different among articles, causing probably significant heterogeneity in our analysis. However, the different laboratory methodologies among the centers might justify this difference. Furthermore, previous drug treatment before admission of COVID-19 was not investigated. Multivariate analysis was not applied; thus, the investigated prognostic factors should not be regarded as independent risk factors. This all could contribute to the significant heterogeneity in some of our results.

### Implication for Practice

The establishment of a prognostic score assessing the possible outcomes of patients suffering from any liver pathology is needed. This meta-analysis succeeded to identify some factors, with high specificity, which might be a footstone for such a prognostic tool that might be completed by additionally recognized risk factors, for example, elevated absolute white blood cell count, decreased lymphocyte count, and elevated interleukin-6 and serum ferritin concentrations (80). Patients who are affected by the underlying liver pathology might need advanced therapy earlier to avoid undesired clinical outcomes.

### Implication for Research

Based on our results and previously published analyses, further basic research is crucial for a better understanding of the liver injury caused by COVID-19, hepatic comorbidities, and treatment itself.

## Conclusion

In conclusion, on-admission platelet count, ALT and AST activities, CRP concentration, and the presence of acute and CLDs predicted the severe course of COVID-19. To highlight, investigating hepatic injury associated by SARS-CoV-2 infection may play an important role in the prediction of mortality and may be used for the establishment of prognostic tools to identify patients with possible poorer outcomes.

## Data Availability Statement

All datasets generated for this study are included in the article/Supplementary Material.

## Author Contributions

SV, PJH, NZ, LS, and NV conceived the study. SV, PHe, and GP wrote the protocol. LS and NZ did the literature search. PJH, NV, ZD, and FD screened the records and extracted the data. KJ, MF, and SK validated the extracted data. PHa and TH assessed the quality of included studies. DN did the statistical analysis. SV, ZS, ZR, and KO prepared the tables. NZ, LS, NV, SV, and PJH wrote the first draft of this manuscript. BE, ZS, GP, and PHe supervised the manuscript and approved the submitted draft. GP is the guarantor of this paper and, as a hepatologist, provided the team with an expert background. All authors provided critical conceptual input, interpreted the data analysis, and critically revised and approved the final version of the manuscript.

## Conflict of Interest

The authors declare that the research was conducted in the absence of any commercial or financial relationships that could be construed as a potential conflict of interest.
