# Supplementary material for: Pre-existing Liver Diseases and On-Admission Liver-Related Laboratory Tests in COVID-19: A Prognostic Accuracy Meta-Analysis With Systematic Review
Source: Front Med (Lausanne). 2020 Nov 13;7:572115. doi: 10.3389/fmed.2020.572115 (PMC7691431; doi:10.3389/fmed.2020.572115)
Supplement: Supplementary file 1 [file Data_Sheet_1.docx]

Supplementary Material

Pre-existing liver diseases and on-admission liver-related laboratory tests in COVID-19: A prognostic accuracy meta-analysis with systematic review.

Authors

Szilárd Váncsa^1,2*^, Péter Jenő Hegyi^1*^, Noémi Zádori^1,2^, Lajos Szakó^1,2^, Nóra Vörhendi^1,2^, Klementina Ocskay^1,2^, Mária Földi^1,2,3^, Fanni Dembrovszky^1,2^, Zsuzsa Réka Dömötör^4^, Kristóf Jánosi^5^, Zoltán Rakonczay Jr.^6^, Petra Hartmann^7^, , Tamara Horváth^7^, Bálint Erőss^1^, Szabolcs Kiss^1,2,3^, Zsolt Szakács^1,2^, Dávid Németh^1^, Péter Hegyi^1^, Gabriella Pár^8^, on behalf of the KETLAK Study Group

*authors contributed equally to the study

**Affiliation**

1. Institute for Translational Medicine, Medical School, University of Pécs, Pécs, Hungary
2. János Szentágothai Research Centre, University of Pécs, Pécs, Hungary
3. Doctoral School of Clinical Medicine, University of Szeged, Szeged, Hungary
4. University of Medicine, Pharmacy, Science and Technology of Targu Mures, Targu Mures, Romania
5. Heart Institute, Medical School, University of Pécs, Pécs Hungary
6. Department of Pathophysiology, University of Szeged, Szeged, Hungary
7. Institute of Surgical Research, University of Szeged, Szeged, Hungary
8. Division of Gastroenterology, First Department of Medicine, Medical School, University of Pécs, Pécs, Hungary

# TABLE OF CONTENTS

**Supplementary Table 1.** PRISMA checklist

**Supplementary Table 2.** Classification of COVID-19 severity

**Supplementary Table 3.** Basic characteristics of included studies – prognostic factors

**Supplementary Table 4.** Eligibility criteria in each included study

**Supplementary Table 5.** Risk of bias assessment using the modified QUIPS tool

**Supplementary Table 6.** On-admission liver related parameter cut-off values

**Supplementary Table 7.** Pre-existing liver diseases definition

**Supplementary Figure 1-3.** Hierarchical summary receiver-operating characteristic (HSROC) curves for each outcome and prognostic factor assessed

**Supplementary Figure 4-17.** Forest plots with prognostic factors on COVID-19 outcomes

**References**

# SUPPLEMENTARY TABLES AND FIGURES

## Supplementary Tables

Supplementary Table 1. PRISMA checklist(1)

| **Section/topic** | **#** | **Checklist item** | **Reported on page #** |
| --- | --- | --- | --- |
| **TITLE** | | |  |
| Title | 1 | Identify the report as a systematic review, meta-analysis, or both. | 1 |
| **ABSTRACT** | | |  |
| Structured summary | 2 | Provide a structured summary including, as applicable: background; objectives; data sources; study eligibility criteria, participants, and interventions; study appraisal and synthesis methods; results; limitations; conclusions and implications of key findings; systematic review registration number. | 1-2 |
| **INTRODUCTION** | | |  |
| Rationale | 3 | Describe the rationale for the review in the context of what is already known. | 2 |
| Objectives | 4 | Provide an explicit statement of questions being addressed with reference to participants, interventions, comparisons, outcomes, and study design (PICOS). | 2 |
| **METHODS** | | |  |
| Protocol and registration | 5 | Indicate if a review protocol exists, if and where it can be accessed (e.g., Web address), and, if available, provide registration information including registration number. | 2 |
| Eligibility criteria | 6 | Specify study characteristics (e.g., PICOS, length of follow-up) and report characteristics (e.g., years considered, language, publication status) used as criteria for eligibility, giving rationale. | 3 |
| Information sources | 7 | Describe all information sources (e.g., databases with dates of coverage, contact with study authors to identify additional studies) in the search and date last searched. | 3 |
| Search | 8 | Present full electronic search strategy for at least one database, including any limits used, such that it could be repeated. | 3 |
| Study selection | 9 | State the process for selecting studies (i.e., screening, eligibility, included in systematic review, and, if applicable, included in the meta-analysis). | 3 |
| Data collection process | 10 | Describe method of data extraction from reports (e.g., piloted forms, independently, in duplicate) and any processes for obtaining and confirming data from investigators. | 3 |
| Data items | 11 | List and define all variables for which data were sought (e.g., PICOS, funding sources) and any assumptions and simplifications made. | 3 |
| Risk of bias in individual studies | 12 | Describe methods used for assessing risk of bias of individual studies (including specification of whether this was done at the study or outcome level), and how this information is to be used in any data synthesis. | 4 |
| Summary measures | 13 | State the principal summary measures (e.g., risk ratio, difference in means). | 3 |
| Synthesis of results | 14 | Describe the methods of handling data and combining results of studies, if done, including measures of consistency (e.g., I^2^) for each meta-analysis. | 3 |
| Risk of bias across studies | 15 | Specify any assessment of risk of bias that may affect the cumulative evidence (e.g., publication bias, selective reporting within studies). | 4 |
| Additional analyses | 16 | Describe methods of additional analyses (e.g., sensitivity or subgroup analyses, meta-regression), if done, indicating which were pre-specified. | - |
| **RESULTS** | | |  |
| Study selection | 17 | Give numbers of studies screened, assessed for eligibility, and included in the review, with reasons for exclusions at each stage, ideally with a flow diagram. | 4 |
| Study characteristics | 18 | For each study, present characteristics for which data were extracted (e.g., study size, PICOS, follow-up period) and provide the citations. | Table 1 |
| Risk of bias within studies | 19 | Present data on risk of bias of each study and, if available, any outcome level assessment (see item 12). | Supplementary Table 5 |
| Results of individual studies | 20 | For all outcomes considered (benefits or harms), present, for each study: (a) simple summary data for each intervention group (b) effect estimates and confidence intervals, ideally with a forest plot. | Supplementary Figure 1-17 |
| Synthesis of results | 21 | Present results of each meta-analysis done, including confidence intervals and measures of consistency. | 4-5 |
| Risk of bias across studies | 22 | Present results of any assessment of risk of bias across studies (see Item 15). | 5 |
| Additional analysis | 23 | Give results of additional analyses, if done (e.g., sensitivity or subgroup analyses, meta-regression [see Item 16]). | - |
| **DISCUSSION** | | |  |
| Summary of evidence | 24 | Summarize the main findings including the strength of evidence for each main outcome; consider their relevance to key groups (e.g., healthcare providers, users, and policy makers). | 5-6 |
| Limitations | 25 | Discuss limitations at study and outcome level (e.g., risk of bias), and at review-level (e.g., incomplete retrieval of identified research, reporting bias). | 6 |
| Conclusions | 26 | Provide a general interpretation of the results in the context of other evidence, and implications for future research. | 6 |
| **FUNDING** | | |  |
| Funding | 27 | Describe sources of funding for the systematic review and other support (e.g., supply of data); role of funders for the systematic review. | 7 |

**Supplementary Table 2.** Classification of COVID-19 severity (2)

| **Severity** | **Definition** |
| --- | --- |
| Mild | The clinical symptoms are mild with no abnormal radiological findings. |
| Moderate | Fever, cough and other symptoms are presented with pneumonia on chest computed tomography. |
| Severe | One of the following conditions is met: (1) Respiratory distress, respiratory rate ≥ 30 per min; (2) Oxygen saturation on room air at rest ≤ 93%; (3) Partial pressure of oxygen in arterial blood / fraction of inspired oxygen ≤ 300 mmHg. |
| Critical | One of the following conditions has to be met: (1) Respiratory failure occurs and mechanical ventilation is required; (2) Shock occurs; (3) Patients with other organ dysfunction needing intensive care unit monitoring. |

Supplementary Table 3. Basic characteristics of included studies – prognostic factors

| **Study** | **TBIL** | **PLT** | **INR** | **Albumin** | **ALT** | **AST** | **LDH** | **CRP** | **ALP** | **GGT** | **CLD** | **LD** | **CBH** | **FLD** | **LF** |
| --- | --- | --- | --- | --- | --- | --- | --- | --- | --- | --- | --- | --- | --- | --- | --- |
| Cai Q et al.(3) |  |  |  |  |  |  |  |  |  |  | + |  |  | + | + |
| Cai Q et al.(4) | + |  |  |  | + | + |  |  | + | + |  |  |  |  | + |
| Cao J et al.(5) |  |  |  |  |  |  |  |  |  |  | + |  |  |  | + |
| Chen G et al.(6) |  | + |  | + |  | + | + |  |  |  |  |  |  |  | + |
| Chen R et al.(7) | + | + |  |  | + | + | + | + |  |  |  |  | + |  |  |
| Chen T et al.(8) |  |  |  | + | + | + | + |  |  |  |  |  | + |  | + |
| Chen TL et al.(9) |  | + |  | + | + | + | + | + |  |  | + |  |  |  |  |
| Chen X et al.(10) |  |  |  |  |  |  |  |  |  |  | + |  |  |  |  |
| Colombi D et al.(11) |  |  |  |  |  |  |  |  |  |  |  | + |  |  |  |
| Du RH et al.(12) |  | + |  | + | + | + |  | + |  |  |  |  |  |  |  |
| Fan BE et al.(13) |  | + |  |  |  |  | + |  |  |  |  |  |  |  |  |
| Fan Z et al.(14) |  |  |  |  |  |  |  |  |  |  |  | + |  |  |  |
| Feng Y et al.(15) |  |  |  |  |  |  |  | + |  |  |  |  |  |  |  |
| Goyal P et al.(16) | + | + |  |  | + | + |  | + |  |  | + |  |  |  |  |
| Grein J et al.(17) |  |  |  |  |  |  |  |  |  |  |  |  |  |  | + |
| Guan W et al.(18) | + | + |  |  | + | + | + | + |  |  |  |  | + |  |  |
| Guan WJ et al.(19) |  |  |  |  |  |  |  |  |  |  |  |  | + |  |  |
| Huang C et al.(20) |  | + |  |  |  | + | + |  |  |  | + |  |  |  |  |
| Ji D et al.(21) | + |  |  |  | + | + |  |  | + | + |  |  | + | + |  |
| Ji D et al.(22) |  |  |  |  |  |  | + |  |  |  |  |  |  |  |  |
| Li X et al.(23) | + | + |  | + | + | + | + | + |  |  |  |  | + |  | + |
| Liu Y et al.(24) |  | + |  |  |  |  |  |  |  |  |  |  |  |  |  |
| Qi X et al.(25) |  |  |  |  |  |  |  |  |  |  |  | + |  |  |  |
| Qian Z et al.(26) |  |  |  |  |  |  |  |  |  |  |  |  | + | + |  |
| Qin C et al.(27) |  |  |  |  |  |  |  |  |  |  | + |  |  |  |  |
| Richardson S et al.(28) |  |  |  |  |  |  |  |  |  |  |  |  |  |  | + |
| Ruan Q et al.(29) |  |  |  |  |  |  |  |  |  |  | + |  |  |  |  |
| Shen L et al.(30) |  |  |  |  |  |  |  |  |  |  | + |  |  |  |  |
| Shi Y et al.(31) |  |  |  |  |  |  |  |  |  |  | + |  |  |  |  |
| To KKW et al.(32) |  | + |  |  | + |  |  |  | + |  |  |  |  |  |  |
| Tu WJ et al.(33) |  |  |  |  |  |  |  | + |  |  |  |  |  |  | + |
| Wan S et al.(34) |  | + |  |  |  | + | + |  |  |  | + |  |  |  |  |
| Wan S et al.(35) |  | + |  |  |  |  |  |  |  |  |  |  |  |  |  |
| Wang L et al.(36) |  |  |  |  |  |  |  |  |  |  | + |  |  |  | + |
| Wang Y et al.(37) |  |  |  |  |  |  |  |  |  |  |  |  | + |  |  |
| Wang Z et al.(38) |  |  |  |  | + | + | + | + |  |  | + |  |  |  |  |
| Wu J et al.(39) |  |  |  |  |  |  |  |  |  |  | + |  |  |  |  |
| Yang AP et al.(40) |  |  |  |  |  |  |  |  |  |  | + | + | + |  |  |
| Yang X et al.(41) |  | + |  |  |  |  |  |  |  |  |  |  |  |  |  |
| Yang X et al.(42) |  |  |  |  |  |  |  |  |  |  |  | + |  |  |  |
| Zhang G et al.(43) |  |  |  |  |  |  |  |  |  |  | + |  |  |  |  |
| Zhang J et al.(44) |  |  |  | + | + | + | + | + |  |  |  |  |  |  |  |
| Zhang JJ et al.(45) |  |  |  |  |  |  |  | + |  |  |  |  |  | + |  |
| Zhang R et al.(46) |  |  |  |  |  |  | + |  |  |  | + |  |  |  |  |
| Zhang Y et al(47) | + |  | + | + | + | + | + | + | + | + |  |  |  |  |  |
| Zheng F et al.(48) | + | + |  |  | + | + | + | + |  |  | + |  |  |  |  |
| Zheng S et al.(49) |  |  |  |  |  |  |  |  |  |  | + |  |  |  |  |
| Zhou F et al.(50) |  | + |  |  | + |  | + |  |  |  |  |  |  |  |  |
| Zhou W et al.(51) |  |  |  |  |  |  |  |  |  |  | + |  |  |  |  |
| Zhou Y et al.(52) | + | + | + | + | + | + |  | + | + | + |  |  | + |  |  |

**ALP**: alkaline phosphatase; **ALT**: alanine aminotransferase; **AST**: aspartate aminotransferase; **CBH**: chronic B hepatitis; **CLD**: chronic liver disease; **CRP**: C-reactive protein; **FLD**: fatty liver disease; **GGT**: gamma glutamyl transferase; **INR**: international normalized ratio; **LD**: liver dysfunction; **LDH**: lactate dehydrogenase; **LF**: liver failure; **PLT**: platelet count; **TBIL**: total bilirubin

Supplementary Table 4. Eligibility criteria in each included study

| **Study** | **Outcome** | **Outcome definition (“verbatim”)** | **Eligibility** |
| --- | --- | --- | --- |
| Cai Q et al.(3) | severe COVID-19 | International guidelines for community-acquired pneumonia | PCR confirmed COVID19 |
| Cai Q et al.(4) | severe COVID-19 | Severe pneumonia was defined by the presence of any of the following conditions: i) significantly increased respiration rate (RR): RR > −30 times/minute; ii) hypoxia: oxygen saturation (resting state) <−93%; iii) blood gas analysis: partial pressure of oxygen/fraction of inspired oxygen (PaO2) /FiO2) < −300 mmHg (millimeters of Mercury); or iv) the occurrence of respiratory or other organ failure that requires intensive care unit (ICU) monitoring and treatment, or shock. | PCR confirmed COVID-19  abnormal liver function test during hospitalization |
| Cao J et al.(5) | mortality | Mortality in the observation period +15 days follow up | PCR confirmed COVID19 |
| Chen G et al.(6) | severe COVID-19 | Guidence for Coronavirus disease 219 (6th edition) by National Health Commission of China | PCR confirmed COVID19 |
| Chen R et al.(7) | mortality | ND | Exclusion: incompelete medical records |
| Chen T et al.(8) | mortality | Mortality in the observation period +15 days follow up | PCR confirmed COVID19 |
| Chen TL et al.(9) | mortality | COVID-19 was defined according to the diagnostic and treatment guidelines for SARS-CoV-2 issued by the Chinese National Health Committee version 3-6 | PCR confirmed COVID19 |
| Chen X et al.(10) | ICU | The “Diagnosis and Treatment of New Coronavirus Pneumonia (6th edition)” issued by the National Health Commission of China | PCR confirmed COVID19 |
| Colombi D et al.(11) | ICU | ND | PCR confirmed COVID19 |
| Du RH et al.(12) | ICU | ND | PCR confirmed COVID19 |
| Fan BE et al.(13) | ICU | ND | PCR confirmed COVID19 |
| Fan Z et al.(14) | mortality ICU | ND | PCR confirmed COVID19 |
| Feng Y et al.(15) | ICU | 1. Mild type The clinical symptoms are mild with no abnormal radiological findings. 2. Moderate type Fever, cough and other symptoms are presented with pneumonia on chest computed tomography. 3. Severe type The disease is classified as severe if one of the following conditions is met: (1) Respiratory distress, respiratory rate ≥ 30 per min; (2) Oxygen saturation on room air at rest ≤ 93%; (3) Partial pressure of oxygen in arterial blood / fraction of inspired oxygen ≤ 300 mmHg. 4. Critical type One of the following conditions has to be met: (1) Respiratory failure occurs and mechanical ventilation is required; (2) Shock occurs; (3) Patients with other organ dysfunction needing intensive care unit monitoring | PCR confirmed COVID19 |
| Goyal P et al.(16) | ICU | An early-intubation strategy with limited use of high-flow nasal cannulae during this period. | PCR confirmed COVID19  adults 18 years of age or older with confirmed Covid-19, Cases were confirmed through reverse-transcriptase– polymerase-chain-reaction assays performed on nasopharyngeal swab specimens |
| Grein J et al.(17) | ICU | IVM: mechanical ventillation and ECMO; noninvasive oxygen support: NIPPV or high-flow supplemental oxygen | Confirmed SARS-CoV-2 infection who had an oxygen saturation of 94% or less while they were breathing ambient air or who were receiving oxygen support creatinine clearance above 30 ml per minute and serum levels of alanine aminotransferase (ALT) and aspartate aminotransferase (AST) less than five times the upper limit of the normal range, and they had to agree not to use other investigational agents for Covid-19. |
| Guan W et al.(18) | severe COVID-19 | The primary composite end point was admission to an intensive care unit (ICU), the use of mechanical ventilation, or death We defined the degree of severity of Covid-19 (severe vs. nonsevere) at the time of admission using the American Thoracic Society guidelines for community-acquired pneumonia.15 | PCR confirmed COVID19  Excluded: incubation periods of less than 1 day |
| Guan WJ et al.(19) | mortality ICU severe COVID-19 | **severe or non-severe** Covid-19 based on the 2007 American Thoracic Society / Infectious Disease Society of America guidelines. The predictive ability of the need for ICU admission and mortality has been validated previously  **severe cases denoted** at least one major criterion (septic shock requiring vasoactive medications, or respiratory failure requiring mechanical ventilation), or at least three minor criteria (respiratory rate being 30 times per minute or greater, oxygen index being 250 or lower, multiple lobe infiltration, delirium or loss of consciousness, blood urea nitrogen level being 20 mg/dl or greater, blood leukocyte count being 4,000 per deciliter or lower, blood platelet count being 100,000 per deciliter or lower, body temperature being lower than 36 degrees, hypotension necessitating vasoactive drugs for maintaining blood pressure). | PCR confirmed COVID19 |
| Huang C et al.(20) | ICU | Of the 41 patients, 13 (32%) were admitted to the ICU because they required high-flow nasal cannula or higher-level oxygen support measures to correct hypoxaemia.  Acute respiratory distress syndrome (ARDS) and shock were defined according to the interim guidance of WHO | PCR confirmed COVID19 |
| Ji D et al.(21) | severe COVID-19 | Progression of illness was defined as development of at least one of the following: respiratory rate >− 30 breaths/min, resting oxygen saturation <−93% and PaO2/FiO2 <− 300 mmHg or worsening of lung CT findings, during the hospitalization period. | Consecutive patients admitted to 2 designated COVID-19 Hospitals in China with confirmed COVID-19 and information on NAFLD status, confermed by PCR |
| Ji D et al.(22) | severe COVID-19 | Progressive group: development of respiratory rate ≥ 30 breaths/min, resting oxygen saturation ≤ 93%, PaO2/FiO2 ≤ 300 mmHg or requirement of mechanical ventilation, worsening of lung CT findings during the observation period | PCR confirmed COVID19  Excluded: Patients presenting with severe COVID-19 were excluded + patient with primary infection by other pathogens, such as bacteria, fungi, other respiratory virus, mycoplasma, or chlamydia |
| Li X et al.(23) | severe COVID-19 | Severe COVID-19 was defined according to 2019 clinical practice guideline from Infectious Diseases Society of America and American Thoracic Society for diagnosis and treatment of adults with community-acquired pneumonia | PCR confirmed COVID19 |
| Liu Y et al.(24) | mortality | ND | PCR confirmed COVID19 |
| Qi X et al.(25) | severe COVID-19 | ND | PCR confirmed COVID19 |
| Qian Z et al.(26) | severe COVID-19 | Mild patients include those with a clinical classification of light and normal, and severe patients include those with a clinical classification of severe and critical. | PCR confirmed COVID19 |
| Qin C et al.(27) | severe COVID-19 | Fifth Revised Trial Version of the Novel Coronavirus Pneumonia Diagnosis and Treatment Guidance: as severe-type: 1. Respiratory distress with the respiratory rate over 30 per minute; 2. Oxygen saturation ≤ 93% in the resting state; 3. Arterial blood oxygen partial pressure (PaO2) / oxygen concentration (FiO2) ≤300mmHg. | PCR confirmed COVID19 |
| Richardson S et al.(28) | mortality | ND | All consecutive patients who were sufficiently medically ill to require hospital admission with confirmed severe acute respiratory syndrome coronavirus 2 (SARS-CoV-2) infection by positive result on polymerase chain reaction testing of a nasopharyngeal sample were included. |
| Ruan Q et al.(29) | mortality | Patients met the discharge criteria if they had no fever for at least 3 days, significantly improved respiratory function, and had negative SARS-CoV-2 laboratory test results twice in succession | PCR confirmed COVID19 |
| Shen L et al.(30) | severe COVID-19 | The severe cases in this study refer to the patients who had enrolled to the intensive care unit (ICU) and received a treatment for more than 3 days, whereas the other confirmed cases were distributed to the mild group. | PCR confirmed COVID19 |
| Shi Y et al.(31) | severe COVID-19 | ND | PCR confirmed COVID19 |
| To KKW et al.(32) | severe COVID-19 | The need for supplemental oxygen, admission to the intensive care unit (ICU), or death | PCR confirmed COVID19 |
| Tu WJ et al.(33) | mortality | in hospital mortality or discharge | PCR confirmed COVID19 |
| Wan S et al.(34) | severe COVID-19 | The severe group had respiratory distress, RR ≥ 30 beats/minute in a resting state, a mean oxygen saturation of ≤93%, and an arterial blood oxygen partial pressure (PaO2)/oxygen concentration (FiO2) ≤ 300 mm Hg. The critical group had respiratory failure and required mechanical ventilation, the occurrence of shock, and the combined failure of other organs that required ICU monitoring and treatment | PCR confirmed COVID19 |
| Wan S et al.(35) | severe COVID-19 | Patients were divided into mild (including normal and mild) and severe (including severe and critical) groups as follows: Mild: mild clinical symptoms and imaging showing no pneumonia. Normal: symptoms of fever, respiratory tract inflammation, imaging showing pneumonia. Severe: meeting any of the following: respiratory distress, respiratory rate (RR) ≥30 breaths/min in the resting state, mean oxygen saturation ≤93%, arterial blood oxygen partial pressure (PaO2)/fraction of inspired oxygen concentration (FiO2) ≤300 mmHg. Critical: respiratory failure requiring mechanical ventilation, shock occurs; combined failure of other organs requiring Intensive Care Unit (ICU) monitoring and treatment. | Inpatients diagnosed with COVID-19 |
| Wang L et al.(36) | mortality | 28 day mortality | PCR confirmed COVID19 |
| Wang Y et al.(37) | severe COVID-19 | Cases were classified as follows: (1) mild, mild clinical symptoms without pneumonia seen at chest computed tomography; (2) ordinary, fever and other respiratory symptoms with pneumonia seen at imaging; (3) severe, respiratory distress, hypoxia (oxygen saturation, ≤93%), or abnormal results of blood gas analysis (PaO2  50 mm Hg); and (4) critical, respiratory failure requiring mechanical ventilation, shock, or other organ failure requiring intensive care unit monitoring and treatment. | Asymptomatic patients who had laboratory-confirmed positive results for SARS-CoV-2 (based on nucleic acid testing of pharyngeal swab samples  Excluded: suspected COVID-19 with symptoms such as fever, cough, fatigue, poor appetite, diarrhea, and headache at admission. |
| Wang Z et al.(38) | ICU | According to the lowest SpO2 records during admission, we divided these patients into two groups: the SpO2≥90% group (n=55) and the SpO2<90% group (n=14). | PCR confirmed COVID19 |
| Wu J et al.(39) | ICU | ND | PCR confirmed COVID19 |
| Yang AP et al.(40) | severe COVID-19 | On the basis of international guidelines for community-acquired pneumonia. | Epidemiology history, Fever or other respiratory symptoms, Typical CT image abnormities of viral pneumonia, and Positive result of RT-PCR for SARS-CoV-2 RNA. Severe patients additionally met at least one of the following conditions: Shortness of breath, RR≥30 times/min, Oxygen saturation (Resting state) ≤93%, or (3) PaO2 / FiO2 ≤300mmHg |
| Yang X et al.(41) | mortality | Discharge or decease by February the 25th | PCR confirmed COVID19 |
| Yang X et al.(42) | mortality | 28-day mortality | PCR confirmed COVID19 |
| Zhang G et al.(43) | severe COVID-19 | ND | PCR confirmed COVID19 |
| Zhang J et al.(44) | mortality | When patients have slight clinical symptoms without imaging findings of pneumonia they are treated as having a mild condition. When patients have fever or respiratory symptoms, they are identified as having a moderate condition. Patients are considered as having a severe condition if they have the following: respiratory distress and a respiratory rate >30 times per minute, fingertip blood oxygen saturation <93% at rest, and partial arterial oxygen pressure (PaO2)/fraction of inspiration oxygen (FiO2) ≤300 mmHg. Patients are regarded as having a critical condition if they have one of the following: respiratory failure requiring mechanical ventilation, shock, and other organ failure requiring ICU treatment. | Consecutive patients diagnosed with COVID-19 |
| Zhang JJ et al.(45) | severe COVID-19 | Severe COVID-19 was designated when the patients had one of the following criteria: (a) respiratory distress with respiratory frequency ≥30/min; (b) pulse oximeter oxygen saturation ≤93% at rest; and (c) oxygenation index (artery partial pressure of oxygen/inspired oxygen fraction, PaO2/FiO2) ≤ 300 mm Hg. | All hospitalized patients clinically diagnosed as "viral pneumonia" based on their clinical symptoms with typical changes in chest radiology, were preliminary involved |
| Zhang R et al.(46) | severe COVID-19 | Severe - (1) respiratory distress with a breathing rate ≥ 30/min; (2) pulse oximeter oxygen saturation ≤ 93% at rest; (3) oxygenation index (artery partial pressure of oxygen/inspired oxygen fraction, PaO2/FiO2) ≤ 300 mmHg; (4) respiratory failure requiring mechanical ventilation; (5) shock; and (6) combined with other organ failure requiring intensive care unit (ICU) monitoring and treatment. | PCR confirmed COVID19 |
| Zhang Y et al(47) | severe COVID-19 | According to the guidelines for the diagnosis and treatment of novel coronavirus (2019-nCoV) infection by the National Health Commission (Trial Version 5) | Confirmed COVID-19 pneumonia  excluded: 4 patients under 14 years of age and 2 patients with chronic Hepatitis B or cirrhosis were excluded |
| Zheng F et al.(48) | severe COVID-19 | ND | PCR confirmed COVID19 |
| Zheng S et al.(49) | severe COVID-19 | Mild cases include non-pneumonia or mild pneumonia. Severe disease refers to dyspnoea, respiratory rate ≥30/ min, blood oxygen saturation ≤93%, partial pressure of arterial oxygen to fraction of inspired oxygen ratio <300, or lung infiltrates >50% within 24 to 48 hours. | For this analysis, we only included patients with viral loads monitored for more than five days in respiratory and stool samples. |
| Zhou F et al.(50) | mortality | Those who died or were discharged between Dec 29, 2019 (ie, when the first patients were admitted), and Jan 31, 2020 | Enrolled all adult inpatients who were hospitalised for COVID-19 and had a definite outcome (dead or discharged) at the early stage of the outbreak. |
| Zhou W et al.(51) | mortality | ICU mortality | corticosteroids therapy (median hydrocortisoneequivalent dose of 400.0mg/day) was instantly initiated after ICU admission |
| Zhou Y et al.(52) | ICU | (1) mild cases, the clinical symptoms are mild and no pneumonia manifestation can be found in imaging (2) ordinary cases, patients have symptoms like fever and respiratory tract symptoms, and pneumonia manifestation can be seen in imaging; (3) severe cases, meeting any of the following: respiratory distress, RR ≥ 30 breaths/min; the oxygen saturation is less than 93% at a rest state; arterial partial pressure of oxygen (PaO2) / oxygen concentration (FiO2) ≤300 mmHg (1 mmHg = O.133 l < Pa), patients with > 50% lesion progression within 24 to 48 hours in pulmonary imaging should be treated as severe cases; and (4) critical cases, meeting any of the following: respiratory failure in which mechanical ventilation is required; shock occurs; complication with another organ failure that requires monitoring and treatment in ICU | Consecutive patients with PCR confirmed COVID19 |

Supplementary Table 5. Risk of bias assessment using the modified QUIPS tool

| **Study** | **Study participation** | **Study attrition** | **Prognostic factor** | | | | | | | | | | | | | | | **Outcome** | | **Study confounding** | **Statistical analysis reporting** |
| --- | --- | --- | --- | --- | --- | --- | --- | --- | --- | --- | --- | --- | --- | --- | --- | --- | --- | --- | --- | --- | --- |
|  |  |  | **TBIL** | **PLT** | **INR** | **Albumin** | **ALT** | **AST** | **LDH** | **CRP** | **ALP** | **GGT** | **CLD** | **Liver dysfunction** | **Hepatitis B** | **FLD** | **Complication** | **Severe COVID-19** | **ICU** |  |  |
| Cai Q et al.(3) |  |  |  |  |  |  |  |  |  |  |  |  |  |  |  |  |  |  |  |  |  |
| Cai Q et al.(4) |  |  |  |  |  |  |  |  |  |  |  |  |  |  |  |  |  |  |  |  |  |
| Cao J et al.(5) |  |  |  |  |  |  |  |  |  |  |  |  |  |  |  |  |  |  |  |  |  |
| Chen G et al.(6) |  |  |  |  |  |  |  |  |  |  |  |  |  |  |  |  |  |  |  |  |  |
| Chen R et al.(7) |  |  |  |  |  |  |  |  |  |  |  |  |  |  |  |  |  |  |  |  |  |
| Chen T et al.(8) |  |  |  |  |  |  |  |  |  |  |  |  |  |  |  |  |  |  |  |  |  |
| Chen TL et al.(9) |  |  |  |  |  |  |  |  |  |  |  |  |  |  |  |  |  |  |  |  |  |
| Chen X et al.(10) |  |  |  |  |  |  |  |  |  |  |  |  |  |  |  |  |  |  |  |  |  |
| Colombi D et al.(11) |  |  |  |  |  |  |  |  |  |  |  |  |  |  |  |  |  |  |  |  |  |
| Du RH et al.(12) |  |  |  |  |  |  |  |  |  |  |  |  |  |  |  |  |  |  |  |  |  |
| Fan BE et al.(13) |  |  |  |  |  |  |  |  |  |  |  |  |  |  |  |  |  |  |  |  |  |
| Fan Z et al.(14) |  |  |  |  |  |  |  |  |  |  |  |  |  |  |  |  |  |  |  |  |  |
| Feng Y et al.(15) |  |  |  |  |  |  |  |  |  |  |  |  |  |  |  |  |  |  |  |  |  |
| Goyal P et al.(16) |  |  |  |  |  |  |  |  |  |  |  |  |  |  |  |  |  |  |  |  |  |
| Grein J et al.(17) |  |  |  |  |  |  |  |  |  |  |  |  |  |  |  |  |  |  |  |  |  |
| Guan W et al.(18) |  |  |  |  |  |  |  |  |  |  |  |  |  |  |  |  |  |  |  |  |  |
| Guan WJ et al.(19) |  |  |  |  |  |  |  |  |  |  |  |  |  |  |  |  |  |  |  |  |  |
| Huang C et al.(20) |  |  |  |  |  |  |  |  |  |  |  |  |  |  |  |  |  |  |  |  |  |
| Ji D et al.(21) |  |  |  |  |  |  |  |  |  |  |  |  |  |  |  |  |  |  |  |  |  |
| Ji D et al.(22) |  |  |  |  |  |  |  |  |  |  |  |  |  |  |  |  |  |  |  |  |  |
| Li X et al.(23) |  |  |  |  |  |  |  |  |  |  |  |  |  |  |  |  |  |  |  |  |  |
| Liu Y et al.(24) |  |  |  |  |  |  |  |  |  |  |  |  |  |  |  |  |  |  |  |  |  |
| Qi X et al.(25) |  |  |  |  |  |  |  |  |  |  |  |  |  |  |  |  |  |  |  |  |  |
| Qian Z et al.(26) |  |  |  |  |  |  |  |  |  |  |  |  |  |  |  |  |  |  |  |  |  |
| Qin C et al.(27) |  |  |  |  |  |  |  |  |  |  |  |  |  |  |  |  |  |  |  |  |  |
| Richardson S et al.(28) |  |  |  |  |  |  |  |  |  |  |  |  |  |  |  |  |  |  |  |  |  |
| Ruan Q et al.(29) |  |  |  |  |  |  |  |  |  |  |  |  |  |  |  |  |  |  |  |  |  |
| Shen L et al.(30) |  |  |  |  |  |  |  |  |  |  |  |  |  |  |  |  |  |  |  |  |  |
| Shi Y et al.(31) |  |  |  |  |  |  |  |  |  |  |  |  |  |  |  |  |  |  |  |  |  |
| To KKW et al.(32) |  |  |  |  |  |  |  |  |  |  |  |  |  |  |  |  |  |  |  |  |  |
| Tu WJ et al.(33) |  |  |  |  |  |  |  |  |  |  |  |  |  |  |  |  |  |  |  |  |  |
| Wan S et al.(34) |  |  |  |  |  |  |  |  |  |  |  |  |  |  |  |  |  |  |  |  |  |
| Wan S et al.(35) |  |  |  |  |  |  |  |  |  |  |  |  |  |  |  |  |  |  |  |  |  |
| Wang L et al.(36) |  |  |  |  |  |  |  |  |  |  |  |  |  |  |  |  |  |  |  |  |  |
| Wang Y et al.(37) |  |  |  |  |  |  |  |  |  |  |  |  |  |  |  |  |  |  |  |  |  |
| Wang Z et al.(38) |  |  |  |  |  |  |  |  |  |  |  |  |  |  |  |  |  |  |  |  |  |
| Wu J et al.(39) |  |  |  |  |  |  |  |  |  |  |  |  |  |  |  |  |  |  |  |  |  |
| Yang AP et al.(40) |  |  |  |  |  |  |  |  |  |  |  |  |  |  |  |  |  |  |  |  |  |
| Yang X et al.(41) |  |  |  |  |  |  |  |  |  |  |  |  |  |  |  |  |  |  |  |  |  |
| Yang X et al.(42) |  |  |  |  |  |  |  |  |  |  |  |  |  |  |  |  |  |  |  |  |  |
| Zhang G et al.(43) |  |  |  |  |  |  |  |  |  |  |  |  |  |  |  |  |  |  |  |  |  |
| Zhang J et al.(44) |  |  |  |  |  |  |  |  |  |  |  |  |  |  |  |  |  |  |  |  |  |
| Zhang JJ et al.(45) |  |  |  |  |  |  |  |  |  |  |  |  |  |  |  |  |  |  |  |  |  |
| Zhang R et al.(46) |  |  |  |  |  |  |  |  |  |  |  |  |  |  |  |  |  |  |  |  |  |
| Zhang Y et al(47) |  |  |  |  |  |  |  |  |  |  |  |  |  |  |  |  |  |  |  |  |  |
| Zheng F et al.(48) |  |  |  |  |  |  |  |  |  |  |  |  |  |  |  |  |  |  |  |  |  |
| Zheng S et al.(49) |  |  |  |  |  |  |  |  |  |  |  |  |  |  |  |  |  |  |  |  |  |
| Zhou F et al.(50) |  |  |  |  |  |  |  |  |  |  |  |  |  |  |  |  |  |  |  |  |  |
| Zhou W et al.(51) |  |  |  |  |  |  |  |  |  |  |  |  |  |  |  |  |  |  |  |  |  |
| Zhou Y et al.(52) |  |  |  |  |  |  |  |  |  |  |  |  |  |  |  |  |  |  |  |  |  |

**ALP**=alkaline phosphatase; **ALT**=alanine aminotransferase; **AST**=aspartate aminotransferase; **CLD**=chronic liver disease; **COVID-19**=Coronavirus disease 2019; **CRP**=C-reactive protein; **FLD**=fatty liver disease; **GGT**=gamma glutamyl transferase; **ICU**=intensive care unit requirement; **INR**=international normalized ratio; **LDH**=lactate dehydrogenase; **N/a**=not applicable; **PLT**=platelet count; **TBIL=**total bilirubin

**Green:** low risk of bias; **yellow:** unclear risk of bias; **red:** high risk of bias; **blank:** not applicable;

***Modified QUIPS tool methods***

Results of the modified QUIPS score. Low risk of bias was provided in the case of statistical analysis reporting domain, because all included articles reported raw data for our analysis. Study attrition was assessed in the case of prospective cohort studies We waived the risk assessment of study confounding. Study participation, prognostic factor and outcome measurement were assessed in every included article.

Study participation measurement: Low risk of bias was provided if study participant selection process and basic characteristics was described. Unclear risk of bias was attribute if these details were not reported.

Prognostic factor measurement: Low risk of bias was given if a clear definition of the prognostic factor was provided. In the case of unclear risk of bias no information about the definition of the prognostic factor was available. Studies, which described a definition not according to the international definitions of prognostic factors were defined as articles of high risk.

Outcome measurement: Low risk of bias was given if a clear definition, according to the accepted guidelines was provided. In the case of unclear risk of bias no information about the definition of the outcome was available. Studies, which described a definition not according to the accepted definitions of outcomes were defined as high risk carrying articles.

Supplementary Table 6. On-admission liver related parameter cut-off values

| **Study** | **Laboratory parameter cut-off** | | | | | | | | | |
| --- | --- | --- | --- | --- | --- | --- | --- | --- | --- | --- |
|  | **TBIL (mmol/L)** | **PLT (x10^9^/L)** | **INR** | **Albumin (g/L)** | **ALT (U/L)** | **AST (U/L)** | **LDH (U/L)** | **CRP (mg/L)** | **ALP (IU/L)** | **GGT (U/L)** |
| Cai Q et al.(3) |  |  |  |  |  |  |  |  |  |  |
| Cai Q et al.(4) | **17.1** |  |  |  | **40** | **40** |  |  | **135** | **49** |
| Cao J et al.(5) |  |  |  |  |  |  |  |  |  |  |
| Chen G et al.(6) |  | **100** |  | **32** |  | **40** | **300** |  |  |  |
| Chen R et al.(7) | **17.1** | **150** |  |  | **40** | **40** | **250** | **10** |  |  |
| Chen T et al.(8) |  |  |  | **32** | **41** | **40** | **350** |  |  |  |
| Chen TL et al.(9) |  | **100** |  | **40** | **50** | **40** | **243** | **10** |  |  |
| Chen X et al.(10) |  |  |  |  |  |  |  |  |  |  |
| Colombi D et al.(11) |  |  |  |  |  |  |  |  |  |  |
| Du RH et al.(12) |  | **100** |  | **40** | **50** | **40** |  | **10** |  |  |
| Fan BE et al.(13) |  | **150** |  |  |  |  | **550** |  |  |  |
| Fan Z et al.(14) |  |  |  |  |  |  |  |  |  |  |
| Feng Y et al.(15) |  |  |  |  |  |  |  | **10** |  |  |
| Goyal P et al.(16) | **17.1** | **150** |  |  | **40** | **40** |  | **10** |  |  |
| Grein J et al.(17) |  |  |  |  |  |  |  |  |  |  |
| Guan W et al.(18) | **17.1** | **150** |  |  | **40** | **40** | **250** | **10** |  |  |
| Guan WJ et al.(19) |  |  |  |  |  |  |  |  |  |  |
| Huang C et al.(20) |  | **100** |  |  |  | **40** | **245** |  |  |  |
| Ji D et al.(21) | **ULN** |  |  |  | **ULN** | **ULN** | **250** |  | **ULN** | **ULN** |
| Ji D et al.(22) |  |  |  |  |  |  |  |  |  |  |
| Li X et al.(23) | **21** | **150** |  | **35** | **40** | **40** | **250** | **10** |  |  |
| Liu Y et al.(24) |  | **125** |  |  |  |  |  |  |  |  |
| Qi X et al.(25) |  |  |  |  |  |  |  |  |  |  |
| Qian Z et al.(26) |  |  |  |  |  |  |  |  |  |  |
| Qin C et al.(27) |  |  |  |  |  |  |  |  |  |  |
| Richardson S et al.(28) |  |  |  |  |  |  |  |  |  |  |
| Ruan Q et al.(29) |  |  |  |  |  |  |  |  |  |  |
| Shen L et al.(30) |  |  |  |  |  |  |  |  |  |  |
| Shi Y et al.(31) |  |  |  |  |  |  |  |  |  |  |
| To KKW et al.(32) |  | **145** |  |  | **53** |  |  |  | **97** |  |
| Tu WJ et al.(33) |  |  |  |  |  |  |  | **10** |  |  |
| Wan S et al.(34) |  | **125** |  |  |  | **40** | **250** |  |  |  |
| Wan S et al.(35) |  | **125** |  |  |  |  |  |  |  |  |
| Wang L et al.(36) |  |  |  |  |  |  |  |  |  |  |
| Wang Y et al.(37) |  |  |  |  |  |  |  |  |  |  |
| Wang Z et al.(38) |  |  |  |  | **35** | **40** | **245** | **8** |  |  |
| Wu J et al.(39) |  |  |  |  |  |  |  |  |  |  |
| Yang AP et al.(40) |  |  |  |  |  |  |  |  |  |  |
| Yang X et al.(41) |  | **125** |  |  |  |  |  |  |  |  |
| Yang X et al.(42) |  |  |  |  |  |  |  |  |  |  |
| Zhang G et al.(43) |  |  |  |  |  |  |  |  |  |  |
| Zhang J et al.(44) |  |  |  | **LLN** | **ULN** | **ULN** | **ULN** | **ULN** |  |  |
| Zhang JJ et al.(45) |  |  |  |  |  |  |  | **3** |  |  |
| Zhang R et al.(46) |  |  |  |  |  |  | **250** |  |  |  |
| Zhang Y et al(47) | **21** |  | **1.15** | **30** | **50** | **40** | **243** | **10** | **120** | **57** |
| Zheng F et al.(48) | **20.5** | **100** |  |  | **40** | **40** | **225** | **8** |  |  |
| Zheng S et al.(49) |  |  |  |  |  |  |  |  |  |  |
| Zhou F et al.(50) |  | **100** |  |  | **40** |  | **245** |  |  |  |
| Zhou W et al.(51) |  |  |  |  |  |  |  |  |  |  |
| Zhou Y et al.(52) | **20.5** | **125** | **1.15** | **40** | **50** | **40** |  | **10** | **125** | **60** |

**ALP**=alkaline phosphatase; **ALT**=alanine aminotransferase; **AST**=aspartate aminotransferase; **CRP=**C-reactive protein; **GGT**=gamma glutamyl transferase; **INR=**international normalized ratio; **LDH**=lactate dehydrogenase; **LLN**=lower limit of normal; **PLT=**platelet count; **TBIL**=total bilirubin; **ULN=**upper limit of normal

**Supplementary Table 7.** Pre-existing liver diseases definition

| **Study** | **Chronic liver disease (CLD)** | **Liver dysfunction** | **Chronic hepatitis B** | **Fatty liver disease (FLD)** | **Liver failure** |
| --- | --- | --- | --- | --- | --- |
| Cai Q et al.(3) | CLD |  |  | NAFLD | Liver injury - an alanine transaminase (ALT) and/or aspartate aminotransferase (AST) higher than 3-fold of the upper limit unit (ULN), or gamma-glutamyl transferase (GGT) and/or total bilirubin (tBil) higher than 2-fold of the ULN |
| Cai Q et al.(4) |  |  |  |  | Liver failure |
| Cao J et al.(5) | CLD |  |  |  | Liver failure |
| Chen G et al.(6) |  |  |  |  | Liver failure |
| Chen R et al.(7) |  |  | Hepatitis B |  |  |
| Chen T et al.(8) |  |  | HBsAg positive |  | Liver failure |
| Chen TL et al.(9) | CLD |  |  |  |  |
| Chen X et al.(10) | CLD |  |  |  |  |
| Colombi D et al.(11) |  | Hepatic failure |  |  |  |
| Du RH et al.(12) |  |  |  |  |  |
| Fan BE et al.(13) |  |  |  |  |  |
| Fan Z et al.(14) |  | Abnormal liver function tests on admission |  |  |  |
| Feng Y et al.(15) |  |  |  |  |  |
| Goyal P et al.(16) | CLD |  |  |  |  |
| Grein J et al.(17) |  |  |  |  | Increased hepatic enzyme (transaminase) |
| Guan W et al.(18) |  |  | HBsAg positive with or without elevated levels ALT or AST |  |  |
| Guan WJ et al.(19) |  |  | Hepatitis B |  |  |
| Huang C et al.(20) | CLD |  |  |  |  |
| Ji D et al.(21) |  |  | HBsAg positive | NAFLD based on hepatic attenuation index |  |
| Ji D et al.(22) |  |  |  |  |  |
| Li X et al.(23) |  |  | Hepatitis B |  | Liver failure |
| Liu Y et al.(24) |  |  |  |  |  |
| Qi X et al.(25) |  | Liver injury on admission |  |  |  |
| Qian Z et al.(26) |  |  | HBsAg positive | FLD |  |
| Qin C et al.(27) | CLD |  |  |  |  |
| Richardson S et al.(28) |  |  |  |  | Elevation in AST or ALT of >15 times upper limit of normal |
| Ruan Q et al.(29) | CLD |  |  |  |  |
| Shen L et al.(30) | CLD |  |  |  |  |
| Shi Y et al.(31) | CLD |  |  |  |  |
| To KKW et al.(32) |  |  |  |  |  |
| Tu WJ et al.(33) |  |  |  |  | Liver enzyme abnormalities |
| Wan S et al.(34) | CLD |  |  |  |  |
| Wan S et al.(35) |  |  |  |  |  |
| Wang L et al.(36) | CLD |  |  |  | Criteria by the international serious adverse event consortium (ISAEC) |
| Wang Y et al.(37) |  |  | Hepatitis B |  |  |
| Wang Z et al.(38) | CLD |  |  |  |  |
| Wu J et al.(39) | CLD |  |  |  |  |
| Yang AP et al.(40) | CLD | Abnormal liver function | Hepatitis b |  |  |
| Yang X et al.(41) |  |  |  |  |  |
| Yang X et al.(42) |  | Liver dysfunction |  |  |  |
| Zhang G et al.(43) | CLD |  |  |  |  |
| Zhang J et al.(44) |  |  |  |  |  |
| Zhang JJ et al.(45) |  |  |  | FLD and abnormal liver function |  |
| Zhang R et al.(46) | CLD |  |  |  |  |
| Zhang Y et al(47) |  |  |  |  |  |
| Zheng F et al.(48) | CLD |  |  |  |  |
| Zheng S et al.(49) | CLD |  |  |  |  |
| Zhou F et al.(50) |  |  |  |  |  |
| Zhou W et al.(51) | CLD |  |  |  |  |
| Zhou Y et al.(52) |  |  | Hepatitis B |  |  |

Empty cells represent articles not reporting on the prognostic factor. Definition of prognostic factors are defined as in the included studies.

**ALT**=alanine aminotransferase; **AST**=aspartate aminotransferase; **CLD**=chronic liver disease; **FLD**=fatty liver disease; **GGT**=gamma glutamyl transferase; **HBsAg**=hepatitis B surface antigen; **NAFLD=**non-alcoholic fatty liver disease; **TBil**=total bilirubin; **ULN**=upper limit of normal;

**Supplementary Figure 1.** Hierarchical summary receiver operating characteristic curves (HSROC) for (A) liver failure, (B) platelet count, (C) ALT and (D) LDH for predicting mortality of COVID-19.

**A**

**B**

**C**

**D**

Supplementary Figure 2. Hierarchical summary receiver operating characteristic curves (HSROC) for (A) chronic liver disease, (B) platelet count, (C)ALT, (D)AST and (E) CRP for predicting intensive care unit requirement of COVID-19.

**A**

**B**

**C**

**D**

**E**

**A**

**B**

**C**

**D**

**E**

**F**

**G**

Supplementary Figure 3. Hierarchical summary receiver operating characteristic curves (HSROC) for (A) chronic liver disease, (B) chronic B hepatitis, (C) platelet count, (D) ALT, (E) AST, (F) LDH and (G) CRP for predicting severe COVID-19 course.

Supplementary Figure 4. Prognostic role of chronic liver disease on mortality, intensive care unit requirement and severe COVID-19.

Supplementary Figure 5. Prognostic role of liver dysfunction on mortality, intensive care unit requirement and severe COVID-19.


Supplementary Figure 6. Prognostic role of chronic B hepatitis on mortality, intensive care unit requirement and severe COVID-19.

Supplementary Figure 7. Prognostic role of fatty liver disease on severe COVID-19.

Supplementary Figure 8. Prognostic role of liver failure on mortality, intensive care unit requirement and severe COVID-19.

Supplementary Figure 9. Prognostic role of increased serum total bilirubin on mortality, intensive care unit requirement and severe COVID-19.

Supplementary Figure 10. Prognostic role of decreased platelet count on mortality, intensive care unit requirement and severe COVID-19.


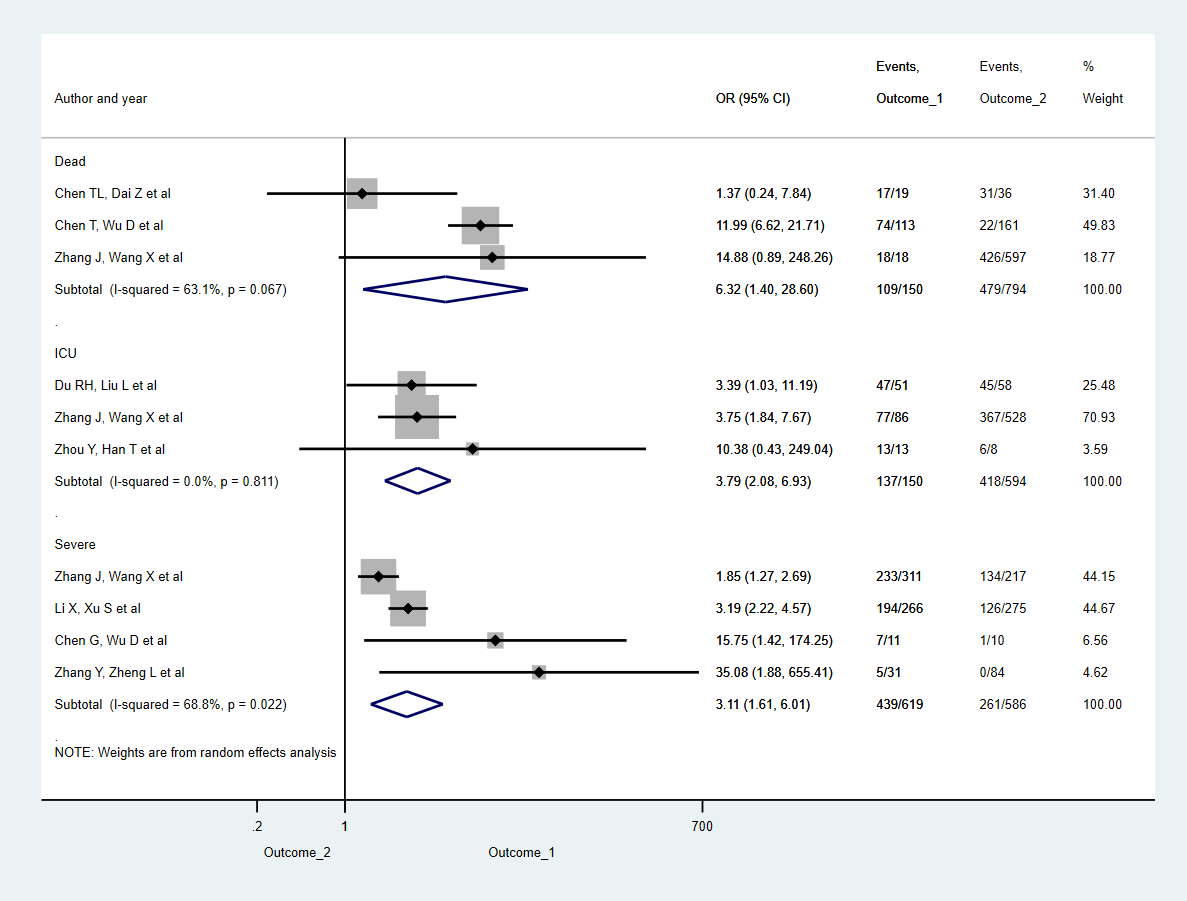


Supplementary Figure 11. Prognostic role of decreased serum albumin on mortality, intensive care unit requirement and severe COVID-19.


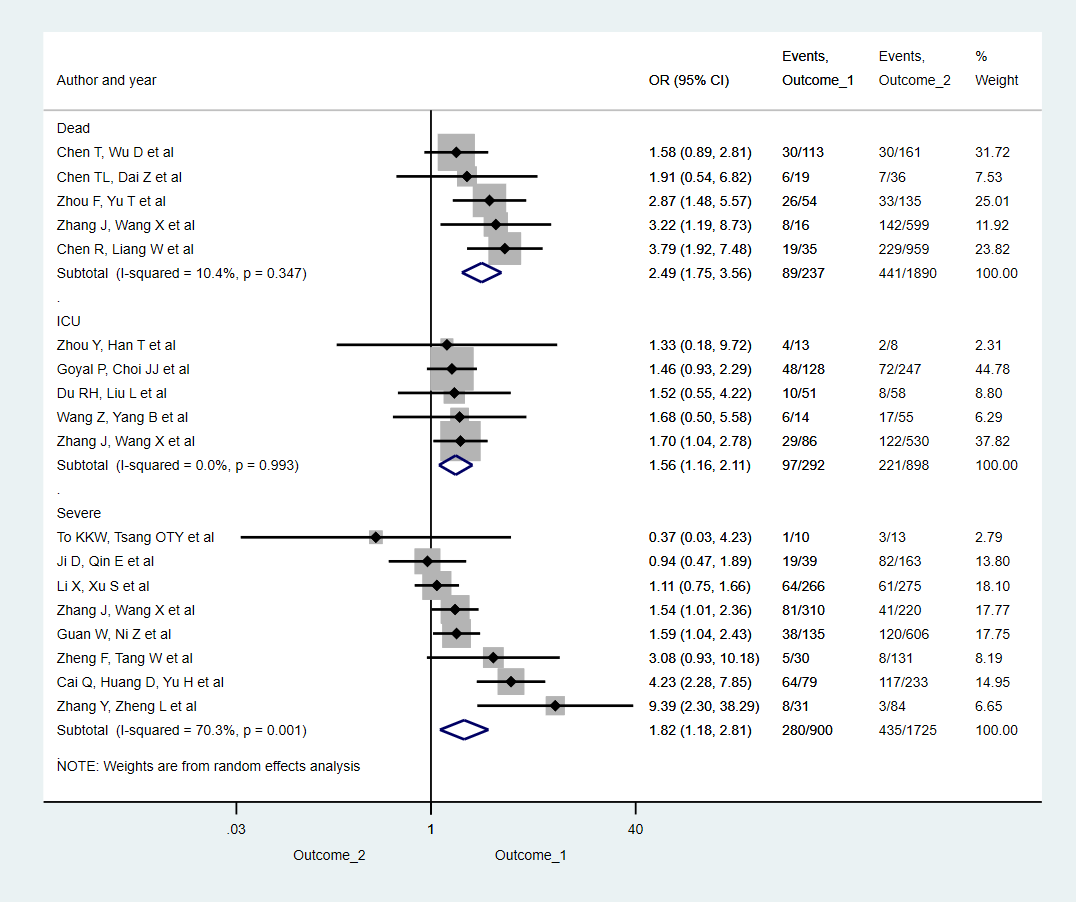


Supplementary Figure 12. Prognostic role of increased alanine aminostransferase on mortality, intensive care unit requirement and severe COVID-19.


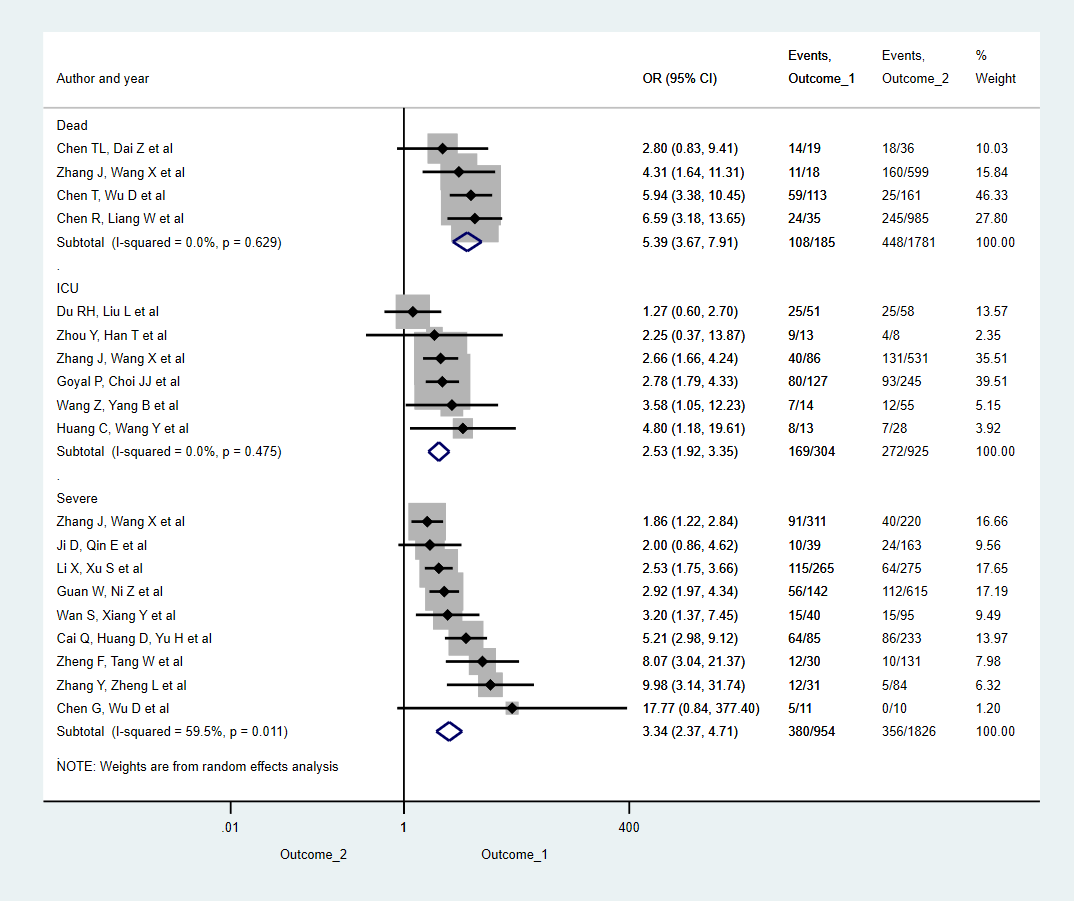


Supplementary Figure 13. Prognostic role of increased aspartate aminotransferase on mortality, intensive care unit requirement and severe COVID-19.


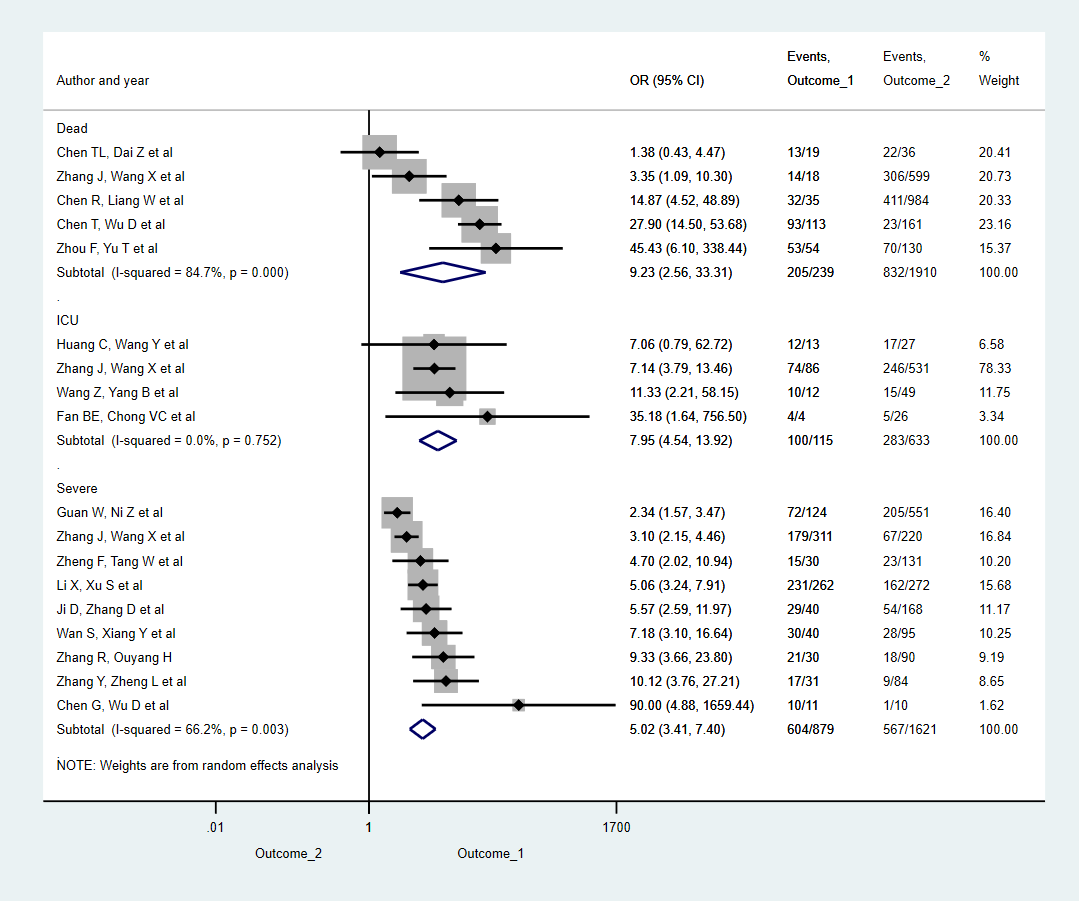


Supplementary Figure 14. Prognostic role of increased lactate dehydrogenase on mortality, intensive care unit requirement and severe COVID-19.


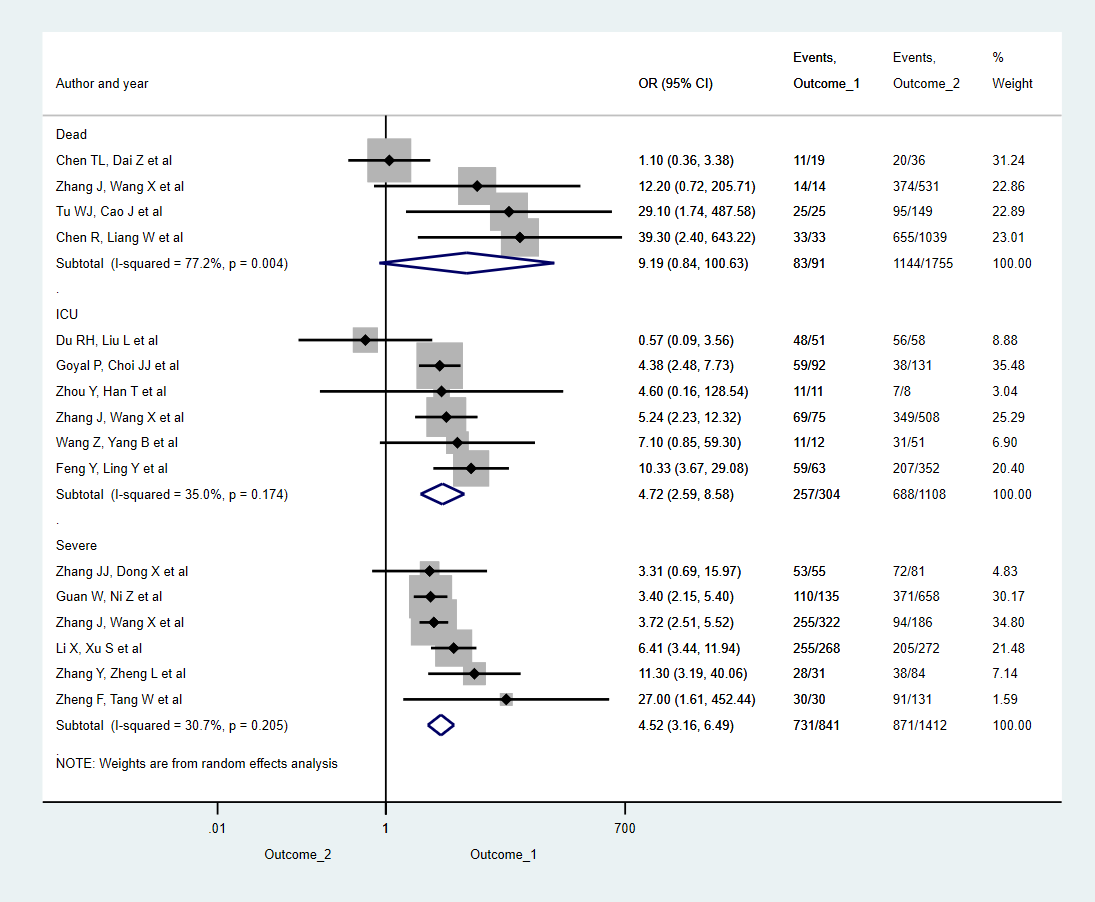


Figure S15. Prognostic role of increased C-reactive protein on mortality, intensive care unit requirement and severe COVID-19.


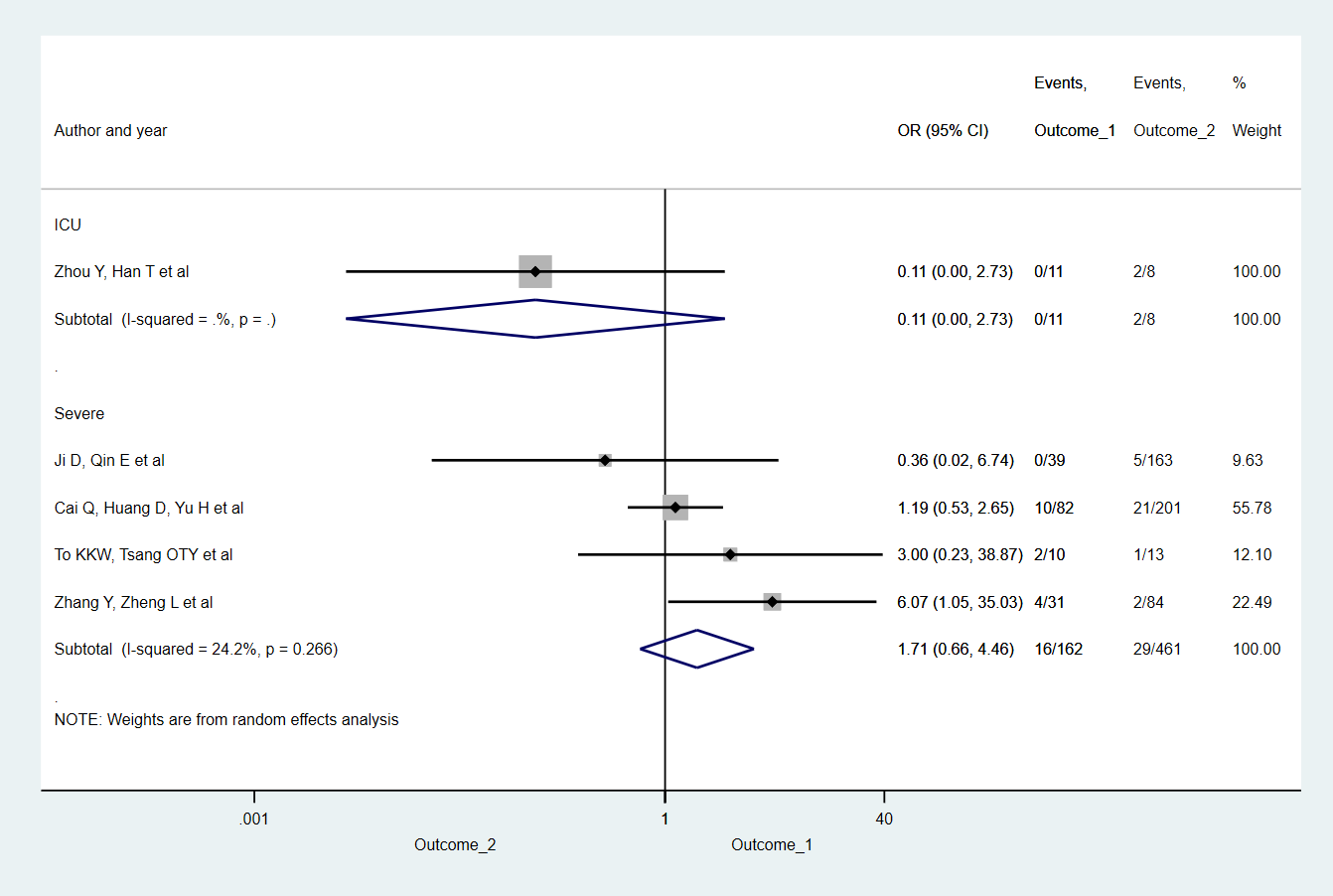
Supplementary Figure 16. Prognostic role of increased alkaline phosphatase on intensive care unit requirement and severe COVID-19.


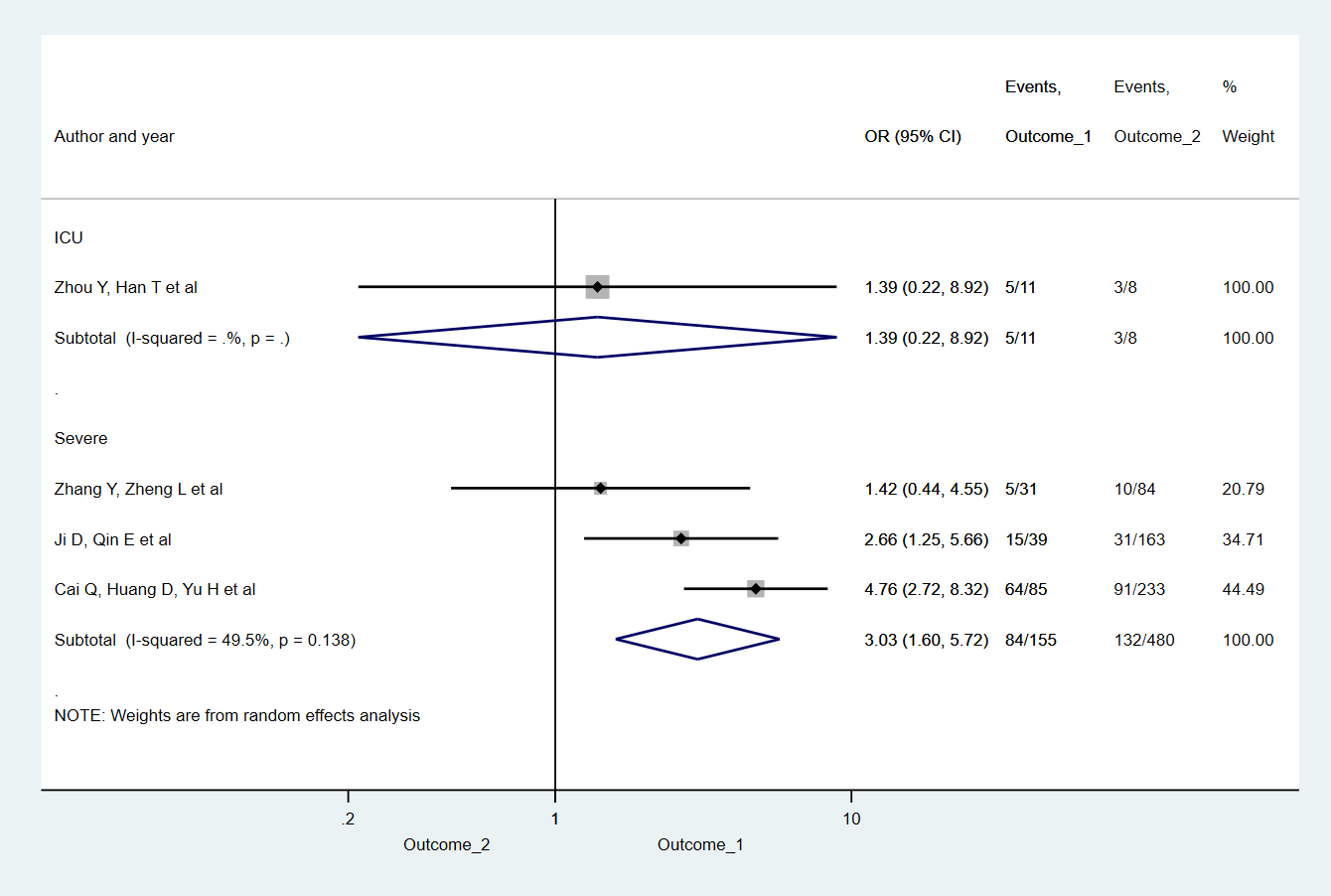
Supplementary Figure 17. Prognostic role of gamma glutamyl transferase on intensive care unit requirement and severe COVID-19.

# REFERENCES

1. Moher D, Liberati A, Tetzlaff J, Altman DG. Preferred reporting items for systematic reviews and meta-analyses: the PRISMA statement. *BMJ*. (2009) 339:b2535. doi: 10.1136/bmj.b2535

2. Zu ZY, Jiang MD, Xu PP, Chen W, Ni QQ, Lu GM, et al. Coronavirus Disease 2019 (COVID-19): A Perspective from China. *Radiology*. (2020) 296(2):E15-E25. doi: 10.1148/radiol.2020200490

3. Cai Q, Huang D, Ou P, Yu H, Zhu Z, Xia Z, et al. COVID-19 in a designated infectious diseases hospital outside Hubei Province, China. *Allergy*. (2020) 75(7):1742-52. doi: 10.1111/all.14309

4. Cai Q, Huang D, Yu H, Zhu Z, Xia Z, Su Y, et al. COVID-19: Abnormal liver function tests. *J Hepatol*. (2020) 73(3):566-74. doi: 10.1016/j.jhep.2020.04.006

5. Cao J, Tu W-J, Cheng W, Yu L, Liu Y-K, Hu X, et al. Clinical Features and Short-term Outcomes of 102 Patients with Corona Virus Disease 2019 in Wuhan, China. *Clin Infect Dis*. (2020) 71(15):748-55. doi: 10.1093/cid/ciaa243

6. Chen G, Wu D, Guo W, Cao Y, Huang D, Wang H, et al. Clinical and immunological features of severe and moderate coronavirus disease 2019. *J Clin Invest*. (2020) 130(5):2620-9. doi: 10.1172/JCI137244

7. Chen R, Liang W, Jiang M, Guan W, Zhan C, Wang T, et al. Risk Factors of Fatal Outcome in Hospitalized Subjects With Coronavirus Disease 2019 From a Nationwide Analysis in China. *Chest*. (2020) 158(1):97-105. doi: 10.1016/j.chest.2020.04.010

8. Chen T, Wu D, Chen H, Yan W, Yang D, Chen G, et al. Clinical characteristics of 113 deceased patients with coronavirus disease 2019: retrospective study. *BMJ*. (2020) 368:m1091. doi: 10.1136/bmj.m1091

9. Chen T, Dai Z, Mo P, Li X, Ma Z, Song S, et al. Clinical characteristics and outcomes of older patients with coronavirus disease 2019 (COVID-19) in Wuhan, China (2019): a single-centered, retrospective study. *J Gerontol*. (2020) 75(9):1788-95. doi: 10.1093/gerona/glaa089

10. Chen X, Zhao B, Qu Y, Chen Y, Xiong J, Feng Y, et al. Detectable serum SARS-CoV-2 viral load (RNAaemia) is closely correlated with drastically elevated interleukin 6 (IL-6) level in critically ill COVID-19 patients. *Clin Infect Dis*. (2020). doi: 10.1093/cid/ciaa449

11. Colombi D, Bodini FC, Petrini M, Maffi G, Morelli N, Milanese G, et al. Well-aerated Lung on Admitting Chest CT to Predict Adverse Outcome in COVID-19 Pneumonia. *Radiology*. (2020) 296(2):E86-E96. doi: 10.1148/radiol.2020201433

12. Du RH, Liu LM, Yin W, Wang W, Guan LL, Yuan ML, et al. Hospitalization and Critical Care of 109 Decedents with COVID-19 Pneumonia in Wuhan, China. *Ann Am Thorac Soc*. (2020) 17(7):839-46. Epub 2020/04/08. doi: 10.1513/AnnalsATS.202003-225OC

13. Fan BE, Chong VCL, Chan SSW, Lim GH, Lim KGE, Tan GB, et al. Hematologic parameters in patients with COVID-19 infection. *Am J Hematol*. (2020) 95(6):E131-E4. doi: 10.1002/ajh.25774

14. Fan Z, Chen L, Li J, Cheng X, Yang J, Tian C, et al. Clinical Features of COVID-19-Related Liver Damage. *Clin Gastroenterol Hepatol*. (2020) 18(7):1561-6. doi: 10.1016/j.cgh.2020.04.002

15. Feng Y, Ling Y, Bai T, Xie Y, Huang J, Li J, et al. COVID-19 with Different Severity: A Multi-center Study of Clinical Features. *Am J Respir Crit Care Med*. (2020) 201(11):1380-8. Epub 2020/04/11. doi: 10.1164/rccm.202002-0445OC

16. Goyal P, Choi JJ, Pinheiro LC, Schenck EJ, Chen R, Jabri A, et al. Clinical Characteristics of Covid-19 in New York City. *N Engl J Med*. (2020) 382(24):2372-4. doi: 10.1056/NEJMc2010419

17. Grein J, Ohmagari N, Shin D, Diaz G, Asperges E, Castagna A, et al. Compassionate Use of Remdesivir for Patients with Severe Covid-19. *N Engl J Med*. (2020) 382(24):2327-36. doi: 10.1056/NEJMoa2007016

18. Guan W-j, Ni Z-y, Hu Y, Liang W-h, Ou C-q, He J-x, et al. Clinical Characteristics of Coronavirus Disease 2019 in China. *N Engl J Med*. (2020) 382(18):1708-20. doi: 10.1056/NEJMoa2002032

19. Guan W-j, Liang W-h, Zhao Y, Liang H-r, Chen Z-s, Li Y-m, et al. Comorbidity and its impact on 1590 patients with Covid-19 in China: A Nationwide Analysis. *Eur Respir J*. (2020) 55(5):2000547. doi: 10.1183/13993003.00547-2020

20. Huang C, Wang Y, Li X, Ren L, Zhao J, Hu Y, et al. Clinical features of patients infected with 2019 novel coronavirus in Wuhan, China. *Lancet*. (2020) 395(10223):497-506. doi: 10.1016/S0140-6736(20)30183-5

21. Ji D, Qin E, Xu J, Zhang D, Cheng G, Wang Y, et al. Non-alcoholic fatty liver diseases in patients with COVID-19: retrospective study. *J Hepatol*. (2020) 73(2):451-3. doi: 10.1016/j.jhep.2020.03.044

22. Ji D, Zhang D, Xu J, Chen Z, Yang T, Zhao P, et al. Prediction for Progression Risk in Patients with COVID-19 Pneumonia: the CALL Score. *Clin Infect Dis*. (2020) 71(6):1393-9. doi: 10.1093/cid/ciaa414

23. Li X, Xu S, Yu M, Wang K, Tao Y, Zhou Y, et al. Risk factors for severity and mortality in adult COVID-19 inpatients in Wuhan. *J Allergy Clin Immunol*. (2020) 146(1):110-8. doi: 10.1016/j.jaci.2020.04.006

24. Liu Y, Sun W, Guo Y, Chen L, Zhang L, Zhao S, et al. Association between platelet parameters and mortality in coronavirus disease 2019: Retrospective cohort study. *Platelets*. (2020) 31(4):490-6. doi: 10.1080/09537104.2020.1754383

25. Qi X, Liu C, Jiang Z, Gu Y, Zhang G, Shao C, et al. Multicenter analysis of clinical characteristics and outcome of COVID-19 patients with liver injury. *J Hepatol*. (2020) 73(2):455-8. doi: 10.1016/j.jhep.2020.04.010

26. Qian ZP, Mei X, Zhang YY, Zou Y, Zhang ZG, Zhu H, et al. [Analysis of baseline liver biochemical parameters in 324 cases with novel coronavirus pneumonia in Shanghai area]. *Zhonghua Gan Zang Bing Za Zhi*. (2020) 28(3):229-33. Epub 2020/04/10. doi: 10.3760/cma.j.cn501113-20200229-00076

27. Qin C, Zhou L, Hu Z, Zhang S, Yang S, Tao Y, et al. Dysregulation of immune response in patients with COVID-19 in Wuhan, China. *Clin Infect Dis*. (2020) 71(15):762-8. doi: 10.1093/cid/ciaa248

28. Richardson S, Hirsch JS, Narasimhan M, Crawford JM, McGinn T, Davidson KW, et al. Presenting Characteristics, Comorbidities, and Outcomes Among 5700 Patients Hospitalized With COVID-19 in the New York City Area. *JAMA*. (2020) 323(20):2052-9. doi: 10.1001/jama.2020.6775

29. Ruan Q, Yang K, Wang W, Jiang L, Song J. Clinical predictors of mortality due to COVID-19 based on an analysis of data of 150 patients from Wuhan, China. *Intensive Care Med*. (2020) 46(5):846-8. doi: 10.1007/s00134-020-05991-x

30. Shen L, Li S, Zhu Y, Zhao J, Tang X, Li H, et al. Clinical and laboratory-derived parameters of 119 hospitalized patients with coronavirus disease 2019 in Xiangyang, Hubei Province, China. *J Infect*. (2020) 81(1):147-78. doi: 10.1016/j.jinf.2020.03.038

31. Shi Y, Yu X, Zhao H, Wang H, Zhao R, Sheng J. Host susceptibility to severe COVID-19 and establishment of a host risk score: findings of 487 cases outside Wuhan. *Crit Care*. (2020) 24(1):108-. doi: 10.1186/s13054-020-2833-7

32. To KK-W, Tsang OT-Y, Leung W-S, Tam AR, Wu T-C, Lung DC, et al. Temporal profiles of viral load in posterior oropharyngeal saliva samples and serum antibody responses during infection by SARS-CoV-2: an observational cohort study. *Lancet Infect Dis*. (2020) 20(5):565-74. Epub 03/23. doi: 10.1016/S1473-3099(20)30196-1

33. Tu W-J, Cao J, Yu L, Hu X, Liu Q. Clinicolaboratory study of 25 fatal cases of COVID-19 in Wuhan. *Intensive Care Med*. (2020) 46(6):1117-20. doi: 10.1007/s00134-020-06023-4

34. Wan S, Xiang Y, Fang W, Zheng Y, Li B, Hu Y, et al. Clinical features and treatment of COVID-19 patients in northeast Chongqing. *J Med Virol*. (2020) 92(7):797-806. Epub 2020/03/22. doi: 10.1002/jmv.25783

35. Wan S, Yi Q, Fan S, Lv J, Zhang X, Guo L, et al. Relationships among lymphocyte subsets, cytokines, and the pulmonary inflammation index in coronavirus (COVID-19) infected patients. *Br J Haematol*. (2020) 189(3):428-37. Epub 2020/04/17. doi: 10.1111/bjh.16659

36. Wang L, He W, Yu X, Hu D, Bao M, Liu H, et al. Coronavirus disease 2019 in elderly patients: Characteristics and prognostic factors based on 4-week follow-up. *J Infect*. (2020) 80(6):639-45. doi: 10.1016/j.jinf.2020.03.019

37. Wang Y, Liu Y, Liu L, Wang X, Luo N, Li L. Clinical Outcomes in 55 Patients With Severe Acute Respiratory Syndrome Coronavirus 2 Who Were Asymptomatic at Hospital Admission in Shenzhen, China. *J Infect Dis*. (2020) 221(11):1770-4. doi: 10.1093/infdis/jiaa119

38. Wang Z, Yang B, Li Q, Wen L, Zhang R. Clinical Features of 69 Cases With Coronavirus Disease 2019 in Wuhan, China. *Clin Infect Dis*. (2020) 71(15):769-77. doi: 10.1093/cid/ciaa272

39. Wu J, Li W, Shi X, Chen Z, Jiang B, Liu J, et al. Early antiviral treatment contributes to alleviate the severity and improve the prognosis of patients with novel coronavirus disease (COVID-19). *J Intern Med*. (2020) 288(1):128-38. doi: 10.1111/joim.13063

40. Yang A-P, Liu J-P, Tao W-Q, Li H-M. The diagnostic and predictive role of NLR, d-NLR and PLR in COVID-19 patients. *Int Immunopharmacol*. (2020) 84:106504-. doi: 10.1016/j.intimp.2020.106504

41. Yang X, Yang Q, Wang Y, Wu Y, Xu J, Yu Y, et al. Thrombocytopenia and its association with mortality in patients with COVID-19. *J Thromb Haemost*. (2020) 18(6):1469-72. doi: 10.1111/jth.14848

42. Yang X, Yu Y, Xu J, Shu H, Xia Ja, Liu H, et al. Clinical course and outcomes of critically ill patients with SARS-CoV-2 pneumonia in Wuhan, China: a single-centered, retrospective, observational study. *Lancet Resp Med*. (2020) 8(5):475-81. doi: 10.1016/S2213-2600(20)30079-5

43. Zhang G, Hu C, Luo L, Fang F, Chen Y, Li J, et al. Clinical features and short-term outcomes of 221 patients with COVID-19 in Wuhan, China. *J Clin Virol*. (2020) 127:104364-. doi: 10.1016/j.jcv.2020.104364

44. Zhang J, Wang X, Jia X, Li J, Hu K, Chen G, et al. Risk factors for disease severity, unimprovement, and mortality in COVID-19 patients in Wuhan, China. *Clin Microbiol Infect*. (2020) 26(6):767-72. doi: 10.1016/j.cmi.2020.04.012

45. Zhang J-j, Dong X, Cao Y-y, Yuan Y-d, Yang Y-b, Yan Y-q, et al. Clinical characteristics of 140 patients infected with SARS-CoV-2 in Wuhan, China. *Allergy*. (2020) 75(7):1730-41. doi: 10.1111/all.14238

46. Zhang R, Ouyang H, Fu L, Wang S, Han J, Huang K, et al. CT features of SARS-CoV-2 pneumonia according to clinical presentation: a retrospective analysis of 120 consecutive patients from Wuhan city. *Eur Radiol*. (2020) 30:4417-26. doi: 10.1007/s00330-020-06854-1

47. Zhang Y, Zheng L, Liu L, Zhao M, Xiao J, Zhao Q. Liver impairment in COVID-19 patients: A retrospective analysis of 115 cases from a single centre in Wuhan city, China. *Liver Int*. (2020) 40(9):2095-103. doi: 10.1111/liv.14455

48. Zheng F, Tang W, Li H, Huang YX, Xie YL, Zhou ZG. Clinical characteristics of 161 cases of corona virus disease 2019 (COVID-19) in Changsha. *Eur Rev Med Pharmacol Sci*. (2020) 24(6):3404-10. Epub 2020/04/10. doi: 10.26355/eurrev_202003_20711

49. Zheng S, Fan J, Yu F, Feng B, Lou B, Zou Q, et al. Viral load dynamics and disease severity in patients infected with SARS-CoV-2 in Zhejiang province, China, January-March 2020: retrospective cohort study. *BMJ*. (2020) 369:m1443. doi: 10.1136/bmj.m1443

50. Zhou F, Yu T, Du R, Fan G, Liu Y, Liu Z, et al. Clinical course and risk factors for mortality of adult inpatients with COVID-19 in Wuhan, China: a retrospective cohort study. *Lancet*. (2020) 395(10229):1054-62. doi: 10.1016/S0140-6736(20)30566-3

51. Zhou W, Liu Y, Tian D, Wang C, Wang S, Cheng J, et al. Potential benefits of precise corticosteroids therapy for severe 2019-nCoV pneumonia. *Signal Transduct Target Ther*. (2020) 5(1):18. doi: 10.1038/s41392-020-0127-9

52. Zhou Y, Han T, Chen J, Hou C, Hua L, He S, et al. Clinical and Autoimmune Characteristics of Severe and Critical Cases with COVID-19. *Clin Transl Sci*. (2020). doi: 10.1111/cts.12805
